# Chemoenzymatic Photoreforming: A Sustainable Approach for Solar Fuel Generation from Plastic Feedstocks

**DOI:** 10.1021/jacs.3c05486

**Published:** 2023-09-06

**Authors:** Subhajit Bhattacharjee, Chengzhi Guo, Erwin Lam, Josephin M. Holstein, Mariana Rangel Pereira, Christian M. Pichler, Chanon Pornrungroj, Motiar Rahaman, Taylor Uekert, Florian Hollfelder, Erwin Reisner

**Affiliations:** † Yusuf Hamied Department of Chemistry, University of Cambridge, Lensfield Road, Cambridge CB2 1EW, U.K.; ‡ Department of Biochemistry, University of Cambridge, Cambridge CB2 1GA, U.K.

## Abstract

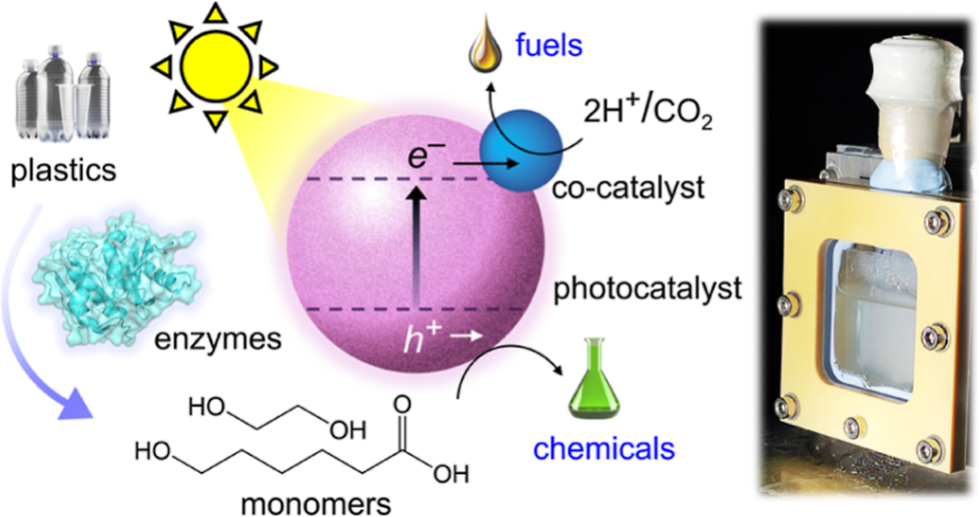

Plastic upcycling through catalytic transformations is an attractive concept to valorize waste, but the clean and energy-efficient production of high-value products from plastics remains challenging. Here, we introduce chemoenzymatic photoreforming as a process coupling enzymatic pretreatment and solar-driven reforming of polyester plastics under mild temperatures and pH to produce clean H_2_ and value-added chemicals. Chemoenzymatic photoreforming demonstrates versatility in upcycling polyester films and nanoplastics to produce H_2_ at high yields reaching ∼10^3^–10^4^ μmol g_sub_^–1^ and activities at >500 μmol g_cat_^–1^ h^–1^. Enzyme-treated plastics were also used as electron donors for photocatalytic CO_2_-to-syngas conversion with a phosphonated cobalt bis(terpyridine) catalyst immobilized on TiO_2_ nanoparticles (TiO_2_|**CotpyP**). Finally, techno-economic analyses reveal that the chemoenzymatic photoreforming approach has the potential to drastically reduce H_2_ production costs to levels comparable to market prices of H_2_ produced from fossil fuels while maintaining low CO_2_-equivalent emissions.

## Introduction

Waste plastics are environmental pollutants,^1,2^ but also represent an untapped chemical resource. Among millions of tons of synthetic plastics generated annually (constituting >30% polyesters^3^), a mere 12% is recycled worldwide.^1,4−6^ The micro- and nanoplastics accumulated in soil and marine biomes are challenging to recycle^7,8^ due to their low concentrations and small sizes.^9−11^ Extracting value from waste plastics requires processes that yield products at low environmental and economic costs.^12^

The discovery of plastic-degrading hydrolases opens a new avenue for polyester depolymerization, by which the enzymatic process can be performed at near-neutral pH (∼6–8) and moderate temperatures (25–65 °C) with low operating costs.^13,14^ Active polyethylene terephthalate (PET) hydrolases have been reported, such as *IsPETase* (obtained from*Ideonella sakaiensis*),^15^ and its more robust variant *DuraPETase* (Dura) with a 300-fold enhanced activity on PET degradation (crystallinity 30%) compared to *IsPETase* at 37 °C.^16^ Enzymatic plastic degradation also demonstrated industrial feasibility with a variant of leaf-compost cutinase (LCC) depolymerizing post-consumer PET waste with low energy input.^17^ However, enzymatic depolymerization has only been demonstrated as a preliminary step for virgin-grade PET regeneration, in which the product range is limiting and of low value. Here, we explore an alternative valorization route to unlock the full potential of enzymatic depolymerization to generate products of greater economic significance from waste plastics.

Photoreforming is emerging as a sustainable approach to utilize and reform depolymerized plastics by harnessing solar energy. The technology enables waste mitigation and the simultaneous generation of valuable fuels and chemicals.^18−20^ Photoreforming typically employs semiconductor photocatalysts that generate electron–hole pairs upon solar irradiation. The photoexcited electrons in the conduction band reduce water to H_2_ and the holes remaining in the valence band oxidize depolymerized plastics into value-added organics.^18,20,21^ The H_2_ formation through photoreforming is a cleaner alternative to current technologies for H_2_ production employing fossil fuel based steam reforming, which releases >800 MtCO_2_ per year.^21,22^ Nonetheless, a major drawback to current plastic photoreforming technologies is the harsh pretreatment conditions (corrosive alkaline media: pH > 13; ∼40–80 °C) required for the depolymerization of plastics.^18,19,21,23^ Other limitations involve low conversion rates and the release of CO_2_ from over-oxidation during photoreforming.^21^ These factors prevent the scaling and commercial considerations of photoreforming despite its potential.

**Figure 1:**
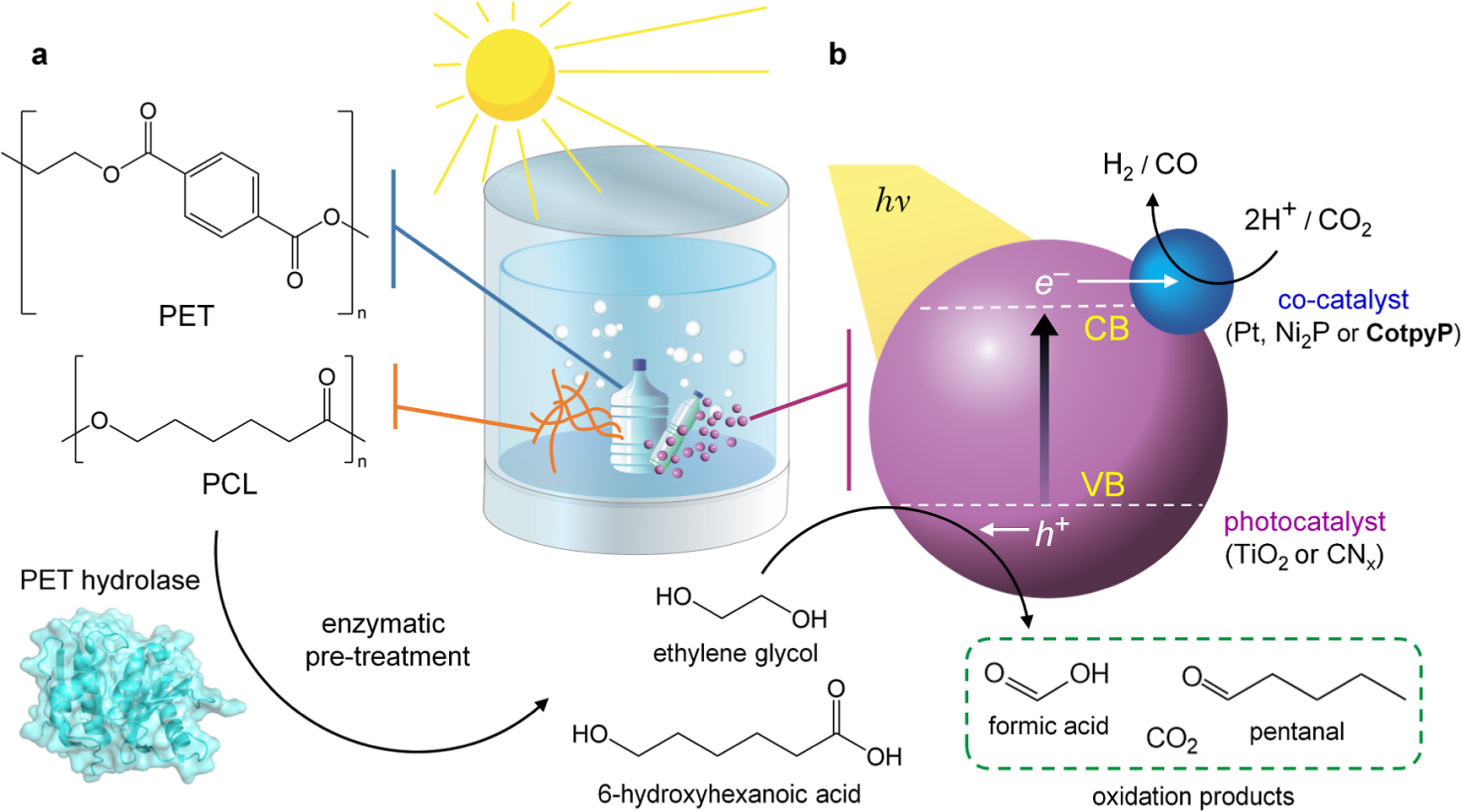
Schematic illustration of the photoreforming process with enzyme pretreatment. (a,b) Illustration of PET and PCL plastics undergoing (a) enzymatic pretreatment in solution followed by (b) photoreforming to yield valuable products. “VB” and “CB” indicate “valence band” and “conduction band”, respectively.

In this work, we introduce chemoenzymatic photoreforming as a process, which combines enzymatic plastic degradation with photoreforming to evolve hydrogen gas from polycaprolactone (PCL) and PET, in the forms of films and nanoplastics, at near-neutral pH (∼6–8) and moderate temperatures (25–65 °C) (Figure 1). Chemoenzymatic photoreforming demonstrated in this work employs two different PET hydrolyses, Dura and LCC, and photocatalysts including Pt-loaded TiO_2_ (TiO_2_|Pt) and Ni_2_P-loaded carbon-nitride (CN_*x*_|Ni_2_P). The overall process produces H_2_ with high yields, surpassing some well-established systems using alkaline pretreatment.^18,19,21^ Chemoenzymatic photoreforming can be performed in two separate steps or in an integrated system, where the two steps are compatible in a one-pot reactor. Furthermore, we demonstrate the utilization of enzyme-treated plastics as a feedstock for photocatalytic CO_2_-to-syngas (H_2_ and CO) production using a TiO_2_|**CotpyP** photocatalyst.^24,25^ Here, apart from reducing externally purged CO_2_, we observe the possibility of converting CO_2_ generated in situ (from over-oxidation of pretreated plastic) to CO.^21^ Finally, our techno-economic analyses for H_2_ production elucidated the economic, environmental, and energetic advantages of combining enzymatic catalysis and photoreforming.^18,21^

## Results and Discussion

### Enzymatic Pretreatment of Plastics

To explore enzymatic degradation at mesophilic and thermophilic temperatures, two PET hydrolases, Dura^16^ and LCC,^17^ were employed for the hydrolysis of PCL and PET (6% crystallinity) films at 37 and 65 °C, respectively. The hydrolysis activity was quantified by high-performance liquid chromatography with ultraviolet absorption (HPLC-UV) measurements of their degradation products: 6-hydroxyhexanoic acid (HA) and terephthalic acid (TPA; produced in equimolar amounts with the oxidizable monomer ethylene glycol (EG) from PET;^19^ as shown in Figure S1 and Movie S1).

At their optimal operating temperatures, LCC (65 °C)^16,17^ yielded 560-fold more TPA than Dura (37 °C)^16^ after a 2-day incubation (Table S1). This observation is consistent with previous reports that at incubation temperatures near the PET glass transition temperature (*T*_g_ ∼67–81 °C)^26^ the enzyme activity increases on degrading the polymer possibly due to increased structural lability resulting in more scissile bonds being accessible for hydrolysis. On a timescale of hours, traces of monomers were detected. Within 2 days of incubation, LCC was observed to be optimal for PET degradation (1120 ± 53 μmol TPA/EG g_sub_^–1^ at 65 °C), whereas Dura was optimal for PCL (56 ± 2 μmol HA g_sub_^–1^ at 37 °C; as shown in Figure S1). Compared to alkaline pretreatment (2 M aqueous NaOH) at identical incubation conditions (i.e., at 37 °C for PCL and 65 °C for PET films) after 2 days on PET and PCL films, higher monomer yields were observed for enzymatic pretreatment on both polyesters (Table S1). Moreover, waste plastics in different sizes are amenable to enzymatic pretreatment. Both LCC and Dura exhibit degradation activity on PET and PCL nanoplastics (⌀ 130–185 nm, NP) with an increase in the mole yield to substrate mass ratio of ∼4-fold for PET and ∼8-fold for PCL after a 2 day incubation compared to those with films (Table S1).

### Photoreforming of Enzyme-Treated Plastics

TiO_2_ nanoparticles (∼20 nm diameter, P25) loaded with a Pt co-catalyst (TiO_2_|Pt-1wt %) and unfunctionalized carbon-nitride loaded with Ni_2_P nanoparticles (CN_*x*_|Ni_2_P-2wt %) were employed as semiconductor powders for the photoreforming of enzyme-treated plastics (see Experimental Section for synthesis details). The loadings of the co-catalyst on the individual photosensitizers (TiO_2_ or CN_*x*_) were determined using inductively coupled plasma optical emission spectrometry (ICP-OES). The solid-state UV–vis spectra in Figure S2a,b show that TiO_2_|Pt absorbs strongly in the UV region (onset: λ_abs_ < 400 nm), whereas CN_*x*_|Ni_2_P also absorbs in the visible region (λ_abs_ < 450 nm). Fourier transform infrared (FT-IR) spectra of TiO_2_|Pt revealed Ti–O vibrational modes between 500–700 cm^–1^ (Figure S2c), whereas for CN_*x*_|Ni_2_P the vibrations appear at 804 cm^–1^ (heptazine core), between 1132 and 1411 cm^–1^ (−CN bending modes), and at ∼2145 cm^–1^ (C=N stretch), as shown in Figure S2d. The powder X-ray diffraction (PXRD) patterns showed the characteristic peaks of anatase and rutile phases (for P25) of the TiO_2_|Pt photocatalyst (Figure S3a), and the peaks corresponding to (100) and (002) phases of carbon nitride for CN_*x*_|Ni_2_P (Figure S3b).^27^ Transmission electron microscopy (TEM) and energy-dispersive X-ray mapping (EDX) confirmed the deposition of the co-catalysts Pt and Ni_2_P on TiO_2_ and CN_*x*_, respectively (Figure S3c,d). The co-catalyst deposition was further attested by X-ray photoelectron spectroscopy (XPS) in the Pt *4f* and Ni *2p* regions of TiO_2_|Pt and CN_*x*_|Ni_2_P photocatalysts, respectively (Figure S4).

**Figure 2:**
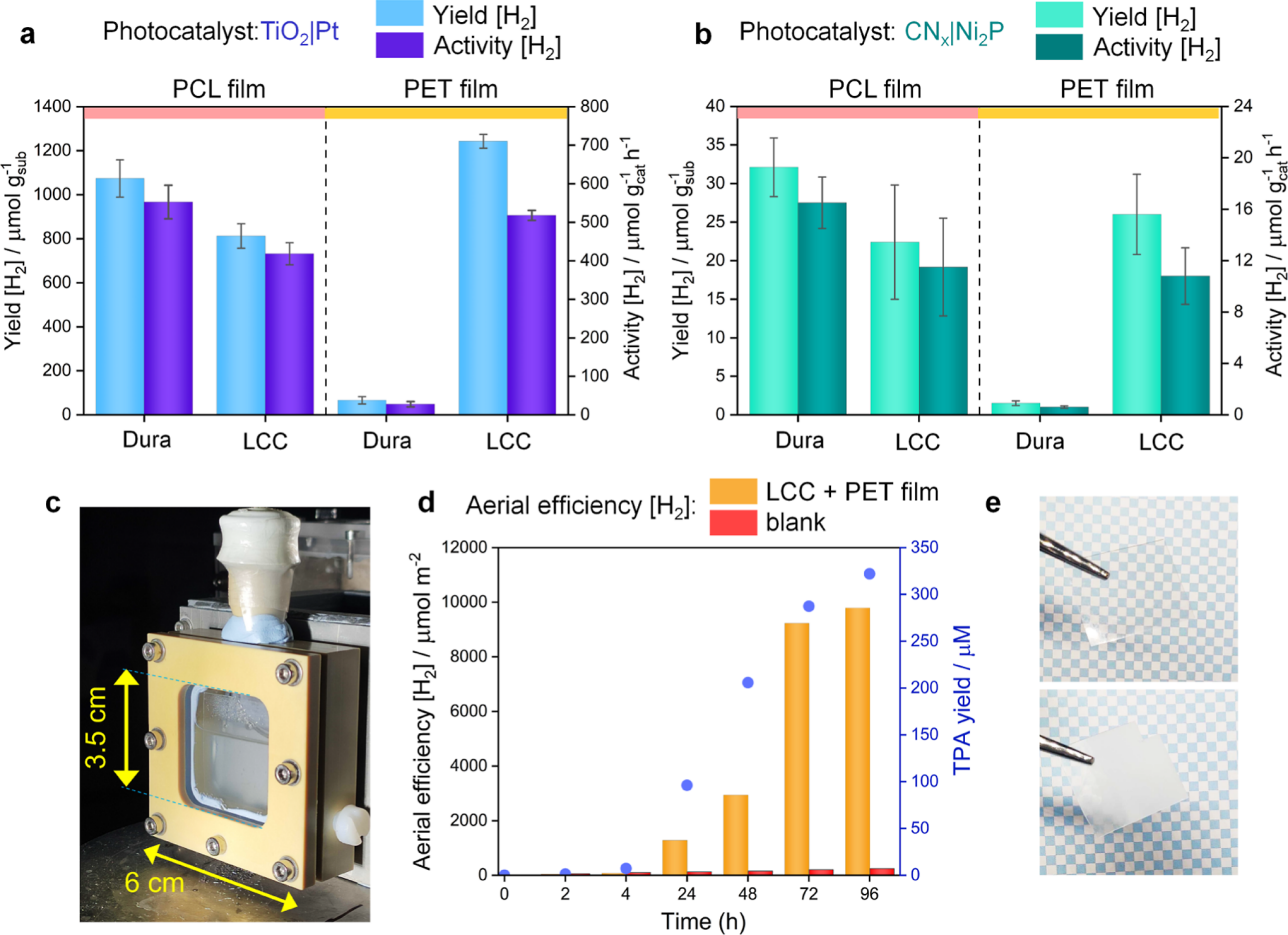
Batch and integrated photoreforming of enzyme-treated plastic films for H_2_ evolution under benign conditions. (a,b) Bar plots showing the yield and activity of H_2_ production from enzyme-treated PCL and PET films employing (a) TiO_2_|Pt and (b) CN_*x*_|Ni_2_P photocatalysts. Conditions: Photocatalyst concentration: 2 mg mL^–1^; carbonate buffer (pH 6); AM 1.5G irradiation; 25 °C; 24 h; stirring. (c) Photograph of the integrated system (with the PET film and LCC enzyme) under operation. (d) Aerial efficiency of H_2_ production and corresponding TPA yield through enzymatic pretreatment over 4 days. During the initial few hours, the rate of PET hydrolysis was negligible as expected considering the limited incubation time. However, after 24 h, a significant increase in the PET hydrolysis (monitored by TPA production) was observed resulting in enhanced H_2_ production. (e) Image of the PET film used for photoreforming before (above) and after (below) four days, showing the change in the texture through enzymatic depolymerization (yielding monomers). Conditions: carbonate buffer (pH 6–8); 33 °C; AM 1.5G irradiation; 96 h incubation without stirring.

The enzymatically pretreated PCL and PET film solutions were first photo-reformed using the semi-heterogeneous photocatalysts in a batch reactor (see the Experimental Section for experimental details) under 1 sun irradiation (AM 1.5G, 100 mW cm^–2^). With the TiO_2_|Pt photocatalyst, the Dura and LCC pretreated PCL films yielded 1074 ± 85 μmol H_2_ g_sub_^–1^ (activity = 553 ± 44 μmol g_cat_^–1^ h^–1^) and 813 ± 56 μmol H_2_ g_sub_^–1^ (activity = 418 ± 29 μmol g_cat_^–1^ h^–1^), respectively (Figure 2a and Table S2). The major corresponding oxidation products identified from the photoreforming of PCL films were pentanal (4.8 ± 0.4 and 2.5 ± 0.2 μmol from Dura and LCC pretreatment, respectively; corresponding activities: 100 ± 8 and 52 ± 4 μmol g_cat_^–1^ h^–1^) and CO_2_ (5.1 ± 0.6 and 2.6 ± 0.4 μmol from Dura and LCC pretreatment, respectively; corresponding activities: 106 ± 13 and 54 ± 8 μmol g_cat_^–1^ h^–1^) with traces of formate and hydrocarbons (pentanal and formate were analyzed using ^1^H nuclear magnetic resonance (NMR) spectroscopy, as shown in Figure S5 and the gaseous CO_2_ and hydrocarbons were determined by GC). Notably, the ∼1:1 ratio of pentanal/CO_2_ provides insights into the mechanism of the solar-driven oxidation reaction (Figure S6). In the first step, the HA formed from the enzymatic pretreatment of PCL films undergoes a 2e^–^ oxidation utilizing the photogenerated holes to form 6-oxohexanoic acid (OA). Thereafter, decarboxylation of OA yields CO_2_ and pentanal in equimolar ratios.

The PET films pretreated with the enzyme Dura did not show an appreciable H_2_ yield (yield: 66 ± 17 μmol g_sub_^–1^; activity: 28 ± 7 μmol g_cat_^–1^ h^–1^) with the TiO_2_|Pt photocatalyst (Figure 2a and Table S3) due to the lower efficiency of Dura to hydrolyze PET at 37 °C as discussed above (see the Enzymatic Pretreatment of Plastics section). However, the LCC enzyme, having higher efficacy toward the PET substrate (at 65 °C) yielded a high concentration of monomers (EG/TPA) and consequently resulted in a higher H_2_ yield (1243 ± 31 μmol g_sub_^–1^) and H_2_ evolution activity (518 ± 13 μmol g_cat_^–1^ h^–1^) with TiO_2_|Pt photocatalysts (Figure 2a and Table S3), higher than other TiO_2_-based systems previously reported.^18,21,28^ The corresponding major oxidation product (produced via EG oxidation) identified after photoreforming was 3.3 ± 0.2 μmol (69 ± 4 μmol g_cat_^–1^ h^–1^) formate (Figure S7).

Next, the CN_*x*_|Ni_2_P photocatalyst was employed for the photoreforming of the pretreated plastic films under similar conditions (Figure 2b and Tables S2 and S3). Although the trend for H_2_ production using CN_*x*_|Ni_2_P was similar to that observed in the case of TiO_2_|Pt, the performance was ∼30–40 times lower and only the oxidation product of PCL photoreforming, i.e., pentanal could be quantified (0.6 ± 0.4 and 0.2 ± 0.05 μmol from Dura and LCC-pretreatment, respectively; Figure S5 and Table S2). The choice of CN_*x*_|Ni_2_P arises from its ability to absorb in the visible region (and for being precious-metal free), as opposed to TiO_2_|Pt, which absorbs primarily in the UV region (Figure S2a,c). This is further confirmed by tests using a λ > 410 nm cut-off filter, which showed that the TiO_2_|Pt was marginally active (retained only ∼0.3% of the activity without filter) toward photoreforming as compared to CN_*x*_|Ni_2_P when using only visible light (Figure S8).

Exclusion control experiments performed by eliminating one component (substrate, enzyme, light, photocatalyst, or co-catalyst) while keeping others fixed did not yield appreciable H_2_ production, as shown in Tables S2 and S3. Additionally, studies without pretreated plastic substrates yielded no formate in the post-catalytic solution, ruling out the possibility of in situ formate production via-photoreduction of the carbonate buffer. External quantum yields (EQY) were determined for TiO_2_|Pt at λ = 360 nm and for CN_*x*_|Ni_2_P at λ = 400 nm (Table S4). With Dura-treated PCL and LCC-treated PET, the EQY for TiO_2_|Pt were 3.6 ± 0.01 and 3.2 ± 0.5%, respectively. The corresponding EQY for CN_*x*_|Ni_2_P were 0.02 ± 0.001% (PCL + Dura) and 0.02 ± 0.004% (PET + LCC).

Following experiments with plastic films, nanoplastics were used for photoreforming under similar conditions. Photoreforming of nanoplastics to generate H_2_ fuel, was so far not considered as a viable option owing to the low concentrations of nanoplastics in aquatic bodies.^8^ Moreover, the utilization of harsh pretreatment conditions (alkaline conditions) commonly employed for plastic photoreforming are not practically feasible in real-world scenarios (e.g., in aquatic bodies such as lakes, oceans, etc.). Photoreforming with varying PET nanoplastics concentrations (0.001, 0.01, 0.1 and 1 mg mL^–1^) shows a steady increase in H_2_ production with comparable activity (Table S3). However, the representative concentration of the nanoplastics used for the pretreatment was chosen to be ∼0.1 mg mL^–1^ as a reasonable upper limit. The H_2_ yields and activities follow a similar trend as for the plastic films with TiO_2_|Pt and CN_*x*_|Ni_2_P and the results are presented in Figure S9 and Tables S2 and S3. The substrate-normalized H_2_ yields are considerably higher (>24,000 μmol g_sub_^–1^ for TiO_2_|Pt and >150 μmol g_sub_^–1^ for CN_*x*_|Ni_2_P; 0.1 mg mL^–1^ substrate concentration) due to the low initial plastic concentrations, with respect to which the amount of H_2_ produced is normalized.

The TiO_2_|Pt system presents the best performing photocatalysts under UV–vis irradiation,^27^ whereas the precious metal-free CN_*x*_|Ni_2_P allows visible-light operation, albeit at lower efficiencies due to limitations from light absorption,^19^ co-catalyst leaching,^19^ oxidative power/kinetics,^29^ and the pH-dependent activity of the co-catalyst.^30^ For example, Ni_2_P is known to perform well as a co-catalyst under alkaline conditions (pH > 13) by forming a thin Ni(OH)_2_ layer facilitating water dissociation, but is less active in neutral pH.^30,31^ From an economics and sustainability perspective, strongly alkaline solutions are not desirable.^21^ Although the use of Pt in the case of TiO_2_ (which is chemically robust, non-toxic and relatively inexpensive) may be considered as a bottleneck for practical applications, we note that the amount of Pt loaded is only 1% and may be sourced from discarded electronic waste materials or other sectors in future and can be reused.^32,33^

The H_2_ evolution activities and yields obtained by our systems using milder enzymatic pretreatment of PCL and PET plastics exceed the values reported for most representative heterogenous photocatalyst systems/processes employing harsh pretreatment conditions^19,21^ as discussed in Table S5.

### Integrated Chemoenzymatic Photoreforming

The photoreforming was next performed in a sealed, custom-made photocatalytic reactor (Figures 2c and S10) to directly couple the enzymatic pretreatment of plastic with the photocatalytic H_2_ generation in an integrated process that facilitates catalyst recovery and continuous processing. For this purpose, the buffer solution consisting of the enzyme and the plastic film was kept between a photocatalyst sheet (prepared by drop-casting a photocatalyst solution on frosted glass; see the Experimental Section for details) and a quartz window, through which the system is illuminated (Figure S10). Photocatalyst sheets are ideally suited as they can be easily mounted, retrieved, and scaled. Moreover, the use of a sheet ensures a clean medium (and not slurries common in heterogenous photocatalysis) for the enzyme to attack the plastics, unperturbed by other components in the suspension such as the photocatalyst in slurries.

The best performing TiO_2_|Pt photocatalyst was used to fabricate the sheet (see the Experimental Section for details), which was then mounted in the reactor. Because PET production and consumption is larger than that of PCL,^34^ a PET film was used for this proof-of-concept demonstration using the integrated system along with the LCC enzyme (see the Experimental Section for details), where the monomers generated from the PET through LCC-pretreatment can be directly utilized during photoreforming using the TiO_2_|Pt sheet.

It was observed that with increasing enzyme-mediated hydrolysis of the PET film, the amount of H_2_ production was also enhanced (Figure 2d and Table S6). After 96 h, the TPA yield was ∼322 μM and the amount of H_2_ produced was 9784 μmol m_irr_^–2^ (∼5 μmol g_cat_^–1^ h^–1^). The plateauing of TPA production indicated deactivation of LCC after 4 days of incubation as also observed in previous studies.^17^ A change in the texture and transparency of the PET film caused by the enzymatic attack was observed after the experiment, as shown in Figure 2e, which was further confirmed using field emission scanning electron microscopy (FESEM) imaging (Figure S11). This further attests that the enzymes are active under the given conditions producing monomers of EG and TPA through PET hydrolysis. Although deconstruction of the entire PET film was not achieved with the enzymes after 96 h, this does not limit the feasibility of the chemoenzymatic reforming process at the industrial scale. In practice, a combination of mechanical and enzymatic approaches can be generally adopted, which leads to ∼70–90% deconstruction of the plastic films within a day.^17^ Moreover, engineering plastic-degrading enzymes will pave the way for improved biological pretreatment systems in the future.

The EG formed from PET can be oxidized by the sheet in situ to produce organics, with the simultaneous generation of H_2_ from the solution. ^1^H NMR spectroscopy of the solution after 96 h of photoreforming confirmed the presence of the oxidation product of EG, formate (5 μmol; Figure S12). Control experiments with blank buffer solutions in the absence of plastics showed negligible H_2_ production after 96 h (Figure 2d and Table S6).

The effect of co-catalyst leaching and generation of organics on the enzyme activity was also studied. ICP-OES analysis revealed Pt co-catalyst leaching to be ∼6% after 24 h. Nonetheless, control experiments were conducted even with larger amounts (i.e., corresponding to the equivalent of 10, 50, and 100% of Pt leached into the buffer solutions) and revealed retention of enzyme activity after 20 h toward PET deconstruction (22 ± 1, 25 ± 5, and 16 ± 2 mM TPA production in buffers corresponding to 10, 50, and 100% Pt co-catalyst leaching, respectively). Additionally, enzymatic reactions in the post-catalytic buffer solution sample used for enzymatic pretreatment (containing organics and very small amount of leached co-catalyst Pt) also suggested that the enzymes remained active toward PET depolymerization under these conditions, producing >50 mM TPA after 20 h of incubation. These results attest the durability of the enzymes under the reaction conditions and their tolerance toward the small amount of metal and organic species present in the solution.

While sequential operation where enzymatic pretreatment is followed by photoreforming provides better control of the individual steps, there are several potential advantages of integrated chemoenzymatic photoreforming from an applied point of view. Integrated operation results in a lower capital and operational cost as it requires only a single combined reactor. It also provides the potential for accelerating the PET breakdown and making the process more energy efficient via heat-management. An integrated system exposes the enzymes to heating from infrared (IR) light from the solar spectrum, which would go to waste in a decoupled process where PET pretreatment occurs in the dark. Thus, our demonstration of photoreforming employing enzyme pretreatment of robust plastic films to generate H_2_ in a single chamber under benign conditions may provide the basis for applied development of this technology.

### Photocatalytic Syngas Production from PET

**Figure 3:**
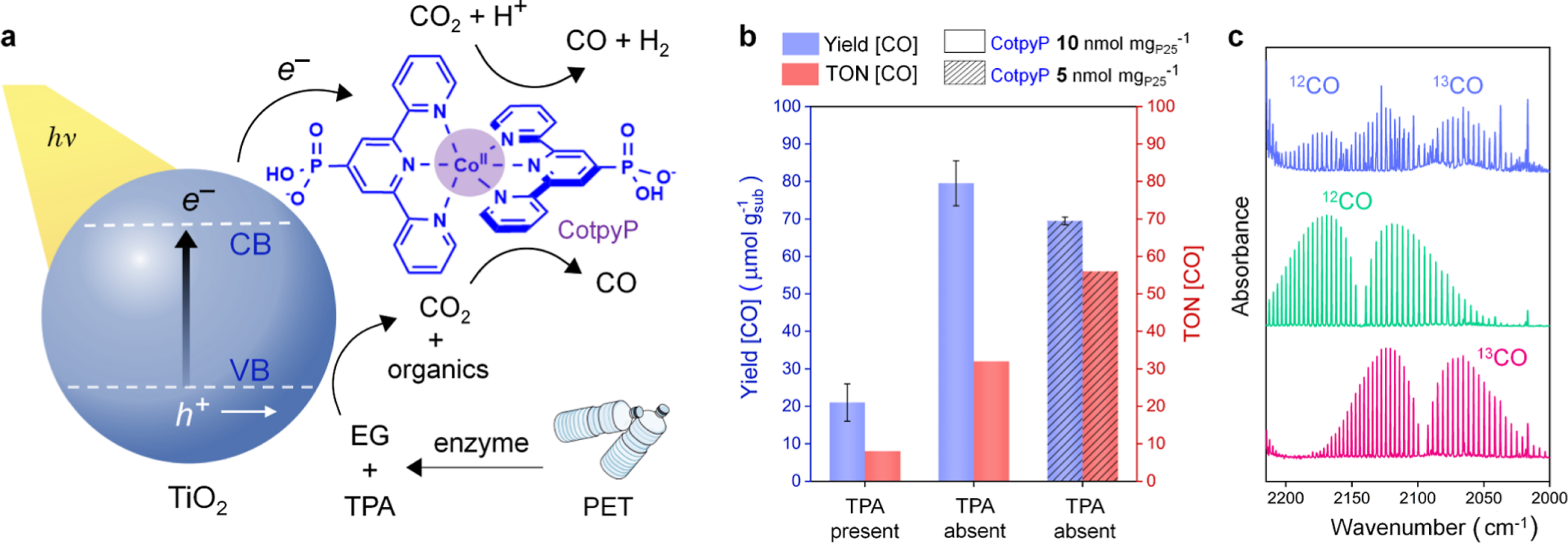
Photocatalytic CO_2_-to-syngas production from enzyme-pretreated PET. (a) Schematic representation of the CO_2_ reduction process using the TiO_2_|**CotpyP** photocatalyst and LCC-treated PET substrate under benign conditions. (b) Bar plots showing the CO yield and corresponding TON for LCC-treated PET films before and after TPA removal and different **CotpyP** loadings. (c) Isotope labeling experiments: gas-phase IR spectra of the headspace (blue) taken after photocatalytic experiments (after TPA removal and pH adjustment; with ^13^CO_2_ saturated, ^13^C-labeled buffer) showing the existence of both ^12^CO and ^13^CO products. The green (^12^CO) and pink (^13^CO) traces are for reference. Conditions for isotopic labeling experiments: MeCN:^13^C-carbonate buffer with LCC-treated PET (2:1); ^13^CO_2_ purging, neutral pH (∼6.5); AM 1.5G irradiation; 48 h at 25 °C with stirring. EG, TPA indicates “ethylene glycol” and “terephthalic acid”, respectively.

The utilization of the greenhouse gas CO_2_ combined with H_2_O for the production of syngas (CO + H_2_) provides a sustainable means to produce an industrially important chemical feedstock.^35^ The pretreatment conditions at near-neutral pH during enzymatic PET hydrolysis enables the utilization of the plastic monomers as an electron donating feedstock for solar-driven CO_2_-to-syngas production (Figure 3a), which is usually performed at near-neutral pH.^36^ Therefore, as a final demonstration, we employ the LCC-treated PET films with a recently reported TiO_2_|**CotpyP** photocatalyst (see the Experimental Section for details) for CO_2_-to-syngas conversion^24^ coupled to plastics oxidation. **CotpyP** is a Co^2+^-based molecular CO_2_ reduction catalyst coordinated by two terpyridine ligands bearing phosphonate groups for anchoring onto TiO_2_.

The photocatalytic reactions with the self-assembled TiO_2_|**CotpyP** (10 or 5 nmol **CotpyP** mg_TiO2_^–1^) were carried out in a mixture of 2:1 MeCN/carbonate buffer (with the carbonate buffer containing the PET monomers). The presence of MeCN shifts the conduction band of TiO_2_ to a more negative potential, facilitating the turnover of the molecular cobalt catalyst.^37^ Only a low yield of CO (21 ± 5 μmol g_sub_^–1^; 1.8 ± 0.4 μmol g_cat_^–1^ h^–1^ after 48 h; as shown in Figure 3b) was observed (with 10 nmol **CotpyP** mg_TiO2_^–1^) in the presence of both EG and TPA (obtained after LCC-pretreatment of PET film) in the medium, suggesting the inhibitory effect of TPA by interfering with the Co-based catalysis (the yields and activity of the accompanied H_2_ evolution reaction are provided in Table S7). Therefore, the reactions were next performed after precipitating TPA from the pretreated solution (by acidification), followed by neutralization (back to pH ∼6.5; clear solution with no residues) to make the pH conducive for the CO_2_ reduction reaction (Figure S13, see the Experimental Section for details). After TPA removal, the yield (80 ± 6 μmol g_sub_^–1^) and activity (7 ± 0.5 μmol g_cat_^–1^ h^–1^; TON_CO_ = 32) for CO production improved significantly after 48 h with 10 nmol **CotpyP** mg_TiO2_^–1^ (Figure 3b). Additionally, on using a lower (5 nmol mg_TiO2_^–1^) **CotpyP** concentration, the TON_CO_ further increased to 56, with no compromise in CO production (with a CO selectivity of ∼20%) and H_2_ as the major reduction product (Figure 3b and Table S7). Control experiments in the absence of photocatalyst or light did not result in product formation (Table S7).

Interestingly, in the absence of externally purged CO_2_, CO production was still observed; although ∼40–50% less as compared to the case with CO_2_ purging (Table S7). Time-dependent evolution plots (using EG and no external CO_2_ purging) in Figure S14 reveal the evolution of CO_2_, but negligible CO production at initial stages. However, after 24 h, the amount of CO increased and exceeded that of the CO_2_ generated in situ. These observations suggest that the TiO_2_|**CotpyP** photocatalyst may also reduce the CO_2_ generated in situ from the overoxidation of EG under the given conditions. Additionally, a non-quantifiable small fraction of CO may also form from partial oxidation/decarbonylation of EG, contributing to the total CO yield.

Isotopic labeling experiments with EG in ^13^CO_2_ purged ^13^C-carbonate buffer (obtained after TPA removal and pH-readjustment; see the Experimental Section for details) revealed the presence of both ^12^CO and ^13^CO after the photocatalytic experiments (Figure 3c). This further corroborates that TiO_2_|**CotpyP** is successful in reducing both the externally purged and in situ generated CO_2_, in addition to other side reactions producing CO. The overall yield of CO is, therefore, a contribution from these individual processes.

These results demonstrate the first example of using plastics as a feedstock for photocatalytic CO_2_-to-syngas production under benign conditions. Moreover, we also observe the possibility of converting the CO_2_ generated in situ (from the over-oxidation of PET monomers) to syngas, thereby alleviating the environmental impacts of photoreforming associated with CO_2_ emissions.

### Techno-Economic Feasibility for H_2_ Production

To understand the commercial feasibility of chemoenzymatic photoreforming, techno-economic analyses were carried out to compare a model chemoenzymatic photoreforming pilot plant with a reported model photoreforming plant with alkaline pretreatment (see Supporting Discussion and Tables S8–S10 for details).^21,38^ The economic and environmental feasibility of the process was estimated using the “base case” and assumed a conversion efficiency of 50% (mol_H_2__ mol_sub_^–1^) and the established metrics: H_2_ production cost (£ kg_H_2__^–1^; both with and without revenue obtained from TPA), carbon footprint (g_CO2_ MJ_H_2__^–1^), and energy returned on energy invested (EROI).^21^

From the techno-economic analyses, the H_2_ production costs, carbon footprint, and EROI obtained with TPA as a co-product are £0.2 kg_H_2__^–1^, 32 g_CO2_ MJ_H_2__^–1^, and 1.5 MJ_H_2__ MJ_input_^–1^ (Tables S8 and S10 and Figure S15), respectively. The corresponding results for the alkaline pretreatment process (using data from previous reports^18^) are £41 kg_H_2__^–1^, 75 g_CO2_ MJ_H_2__^–1^ and 1.3 MJ_H_2__ MJ_input_^–1^ (Tables S9 and S10). These estimates indicate that the enzymatic pretreatment holds the potential to significantly reduce the production costs for green H_2_ (by ∼80–90% compared to alkaline pretreatment if conversion efficiencies can be high) to a price comparable to that of gray hydrogen^39^ while maintaining carbon emissions low.

## Conclusions

The clean and efficient process of utilizing enzymatic pretreatment for photoreforming of polyester plastics (PET and PCL films/nanoplastics) allows us to address two challenges: removing plastic waste from the environment and supplying fuel in the form of hydrogen. Chemoenzymatic photoreforming promises a potential technological solution for converting waste into desirable energy carriers, thereby building a sustainable economy model based on a cost-effective approach for solar-driven fuel/chemical production using waste plastic feedstocks without high energy and non-regenerative reagent consumptions. The process involves enzymes and reusable photocatalysts to produce H_2_ with high yields and activities under mild conditions. We also expand the scope of enzymatic waste pretreatment to photocatalytic CO_2_-to-syngas production using plastic feedstocks in the presence of a Co-based molecular co-catalyst, where the conversion of in situ generated CO_2_ to CO was also observed. Finally, comparative techno-economic analyses for chemoenzymatic photoreforming estimate the H_2_ production costs to be potentially commercially competitive and suggests that this technology has an opportunity to succeed in the marketplace as a waste-to-energy recycling route in supporting the energy transition toward a circular economy.

## Experimental Section

### Materials

P25 TiO_2_ nanoparticles (21 nm, Evonik), chloroplatinic acid solution (H_2_PtCl_6_, 8% v/v, Sigma-Aldrich), trisodium citrate dihydrate (Sigma-Aldrich), sodium borohydride (NaBH_4_, 99%, Sigma-Aldrich), melamine (99%, Sigma-Aldrich), nickel(II) chloride hexahydrate (NiCl_2_·6H_2_O, 99.9%, Sigma-Aldrich), sodium hypophosphite monohydrate (NaH_2_PO_2_·H_2_O, Fischer Scientific), Nafion solution (5 wt %, Sigma-Aldrich), sodium carbonate (Na_2_CO_3_, 99.9%, Sigma-Aldrich), sodium bicarbonate (NaHCO_3_, 99.9%, Sigma-Aldrich), sodium chloride (NaCl, 99.9%, Sigma-Aldrich), acetonitrile (MeCN, Sigma-Aldrich), ^13^C-sodium bicarbonate (NaH^13^CO_3_, 99.9%, Sigma-Aldrich), PET film (Goodfellow, ES30-FM-000125), CO_2_ with 2% CH_4_ (BOC), N_2_ with 2% CH_4_ (BOC), and ^13^CO_2_ (Sigma-Aldrich).

### DuraPETase Expression and Purification

The amino acid sequences for the enzymes are shown in Figure S16. The DuraPETase gene, with a C-terminal Strep-tag, was obtained as a synthetic gene (Thermo Fischer) before cloning into the *Escherichia coli* expression vector pHAT5^40^ with restriction enzymes (Thermo Fischer FastDigest) NcoI and XhoI. For the expression and purification of DuraPETase (Figure S17), single colonies from *E. coli* OverExpress C41 (DE3) (Lucigen) transformations were grown in MDAG-135 non-inducing media^41^ supplemented with 50 μg mL^–1^ carbenicillin (37 °C; 200 rpm shaking; 24 h) as a starter culture. ZYM-5052 media was inoculated with the starter culture (1:100 v/v inoculation ratio) and incubated at 20 °C with 300 rpm shaking until growth saturation was reached for overexpression.

Cells were pelleted by centrifugation at 4000 *xg* for 10 min at 4 °C and resuspended in Buffer W [100 mM Tris (pH 8.0), 150 mM NaCl] before homogenization using Emulsiflex C5. The lysate was cleared by centrifugation at 20,000*xg* for 30 min and passed through a Strep-Tactin (IBA) gravity column following the producer’s protocol for purifying Strep-tag II fusion proteins.^42^ The purified DuraPETase was buffer exchanged into carbonate buffer [27 mM Na_2_CO_3_, 90 mM NaHCO_3_ (pH 8.5), 100 mM NaCl] using PD-10 desalting columns (GE Healthcare) and stored at 4 °C for up to 4 days.

### LCC Expression and Purification

The LCC gene was cloned in pExp-Bla plasmids and transformed into Shuffled T7 express cells (New England BioLabs, catalogue number C3029J). The cells were grown at 37 °C in 1 L of LB media containing 100 μg mL^–1^ ampicillin until the OD_600nm_ reached 0.5 to 0.6. The expression of the recombinant protein was induced by adding 0.4 mM of IPTG at 20 °C for 20 h.

Cells were harvested by centrifugation at 3434 *xg* for 20 min and resuspended in 20 mL of 50 mM Tris–HCl pH 8.0 and lysed via Emulsiflex (Avestin). To the extract were added NaCl, imidazole, and β-mercaptoethanol for a final concentration of 250, 10, and 10 mM, respectively. After lysis, cells were centrifuged at 11,000 *xg* for 45 min at 4 °C to remove cell debris and the supernatant was loaded onto a nickel affinity resin (catalogue number Super-NiNTA25, Protein Ark), previously equilibrated with buffer [50 mM Tris–HCl pH 8.0, 250 mM NaCl, 10 mM imidazole and 5% (v/v) glycerol]. The purification was performed in a stepwise imidazole gradient and the purification fractions analyzed in an SDS-PAGE gel (Figure S18). The fractions containing purified LCC-pExp-Bla recombinant protein were selected for the concentration with Amicon Ultra-15 filters (Merck-Millipore, catalogue number UFC901024). To the concentrated material was added buffer containing 50 mM Tris–HCl pH 8.0, 100 mM NaCl, 2.5% (v/v) glycerol, and the material was submitted to another concentration step. This concentration and dilution step was repeated three times in order to remove the imidazole. To the final concentrated material was added 0.1 mg of TEV protease for cleavage overnight and the material was submitted to a centrifugation at 11,000 *xg* for 15 min at 4 °C to remove any precipitated protein. The supernatant loaded into a column containing a resin previously equilibrated with buffer containing 100 mM NaCl and 100 mM bicarbonate pH 8 for a second purification step via IMAC.

### Preparation of PCL Films

PCL films were generated by dissolving 200 mg of PCL flakes (average *M*_w_ ∼ 14,000, average *M*_n_ ∼ 10,000 by GPC) in 10 mL dichloromethane. 500 μL or 1 mL of the solution were evaporated in an open 1.5 mL tube at 86 °C.

### Preparation of PET and PCL Nanoparticles

The plastic nanoparticles were prepared following a precipitation and solvent evaporation technique as previously described.^43^ Briefly, 50 mg amorphous PET film (product code ES303015, Goodfellow GmbH, London, UK) or PCL flakes were dissolved in 1,1,3,3,3,-hexafluoro-2-propanol (5 mL) for at least 1 h. This solution was added dropwise (1 mL min^–1^) to MilliQ water (50 mL, cooled in an ice bath). At the same time, the water was rigorously stirred using an Ultra Turrax stirrer at 8000 rpm (IKA, Germany). The suspension was filtered using Whatman filter paper (8 μm diameter) and the remaining solvent was evaporated. Particle sizes of *d* = 131.7 nm for PET and *d* = 184.9 nm for PCL were obtained using dynamic light scattering (Zetasizer Nano S).

### Enzymatic Pretreatment

The enzyme (Dura or LCC) stock solutions were spun at 14,000 *xg* (4 °C) for 10 min to eliminate protein precipitation. The protein concentration in the supernatant was determined by measuring absorbance at 280 nm using a Nanodrop ND-1000 spectrophotometer (Nanodrop Technologies). The supernatant was diluted to a concentration of 1 μM with carbonate buffer (see the composition above), and 1 mL of diluted enzyme solution was incubated with either PCL or PET (films or nanoplastics) for 2 days. The incubation temperature for Dura and LCC were 37 and 65 °C, respectively. Prior to the photoreforming tests, the solutions were centrifuged at 20,000 *xg* for 10 min to remove any solid residues from the solution.

### Synthesis of Photocatalysts

The TiO_2_|Pt photocatalyst was prepared by solution-processed platinization of P25 TiO_2_ nanoparticles (Evonik, anatase/rutile, 21 nm) as discussed in previous reports.^27^ Briefly, 150 mg of TiO_2_ was dispersed in 10 mL of MilliQ water through bath sonication for 30 min. Thereafter, 0.29 g of trisodium citrate dihydrate was added to the dispersion followed by sonication for another 30 min. 42 μL of H_2_PtCl_6_ solution (8% in water) was then added to the mixture. After sonication for further 20 min, a freshly prepared NaBH_4_ solution (5 mg dissolved in 1 mL of MilliQ water) was added to the solution dropwise under stirring. After stirring for 30 min, the TiO_2_|Pt photocatalyst was isolated using centrifugation, washed with water, and dried at 80 °C overnight under air.

The CN_*x*_|Ni_2_P photocatalyst was prepared according to previous literature protocols with minor modifications.^19^ Briefly, unfunctionalized carbon nitride (CN_*x*_) was first prepared by heating 2 g of melamine to 550 °C under air for 4 h (ramping rate 5 °C min^–1^) in a covered crucible. 300 mg of the as-prepared CN_*x*_ was then mixed with NiCl_2_·6H_2_O (20 mg for 2 wt %) in minimum volume of MilliQ water (1 mL), followed by stirring and sonication for 1 h each. NaH_2_PO_2_·H_2_O was then added to the reaction mixture and again stirred for 1 h, followed by bath sonication for another 1 h. The mixture was dried in vacuo at 60 °C and the dry solid obtained was heated at 200 °C for 1 h under an Ar atmosphere (ramping rate 5° min^–1^). The CN_*x*_|Ni_2_P powder obtained after cooling to room temperature was washed with ethanol and water and dried in vacuo at 60 °C.

The TiO_2_|**CotpyP** catalyst for CO_2_-to-syngas production was synthesized via an in situ immobilization approach. Briefly, the **CotpyP** molecular catalyst was first prepared according to previously reported protocols,^25^ followed by immobilization with TiO_2_ during photocatalysis (see below).

### Material Characterization

Solid-state UV–vis spectra for the photocatalysts were recorded using a Varian Cary 50 UV–vis spectrophotometer equipped with a diffuse reflectance accessory. FT-IR spectra of the samples were collected using a Thermo Scientific Nicolet iS50 FTIR spectrometer (ATR mode). The PXRD measurements were conducted using a PANalytical Empyrean Series 2 instrument using Cu Kα irradiation. Transmission electron microscopy (TEM) images were acquired using a Thermo Scientific (FEI) Talos F200X G2 TEM. For the TEM measurements, the samples were dispersed in ethanol (∼4 μg mL^–1^) and drop-casted on carbon-coated Cu grids. The FESEM images were acquired using a TESCAN MIRA3 FEG-SEM instrument. The ICP-OES measurements were performed by a Microanalysis Service (Yusuf Hamied Department of Chemistry, University of Cambridge) using a Thermo Scientific iCAP 700 spectrometer. XPS measurements of the photocatalysts were performed at the Maxwell Centre, University of Cambridge with a near ambient pressure (NAP) XPS system with a SPECS XR 50 MF X-ray source, μ-FOCUS 600 X-ray monochromator and a differentially pumped PHOIBOS 150 1D-DLD NAP analyzer. ^1^H NMR spectra were recorded on a 400 MHz Bruker DPX spectrometer and referenced against the residual solvent signal (H_2_O: δ = 4.79 ppm).

### Photoreforming of Enzyme Pretreated Plastics

For the batch studies, 2 mg of the photocatalyst powder (TiO_2_|Pt or CN_*x*_|Ni_2_P) was added to 1 mL of the enzyme pretreated plastic solution (pH ∼6–8) in Pyrex glass photoreactor vials (internal volume: 7.91 mL) and sealed with a rubber septum. The photocatalyst was dispersed via bath sonication for 25 min. Thereafter, the samples were purged with N_2_ (with 2% CH_4_ as an internal standard and leakage control during gas analysis) for another 25 min. The samples were then irradiated using a solar light simulator (Newport Oriel) calibrated to 100 mW cm^–2^ (1 Sun) and equipped with an air mass 1.5 global (AM 1.5 G) filter and a water filter to remove infrared radiation. The temperature was maintained at 25 °C and the samples were stirred at 600 rpm during irradiation. For control experiments involving visible light, a λ > 410 nm cut-off filter was used. The H_2_ generation was monitored by analyzing the reactor headspace gas (50 μL) using gas chromatography (GC) after 24 h (discussed below).

### Chemoenzymatic Photoreforming in Integrated System

As a first step, TiO_2_|Pt photocatalyst sheets were prepared by a modified literature method.^27^ Frosted glass substrates (4.5 × 4.5 cm^2^) were cleaned by sonication in MilliQ water, isopropanol, and acetone, 15 min in each, and then dried under gentle N_2_ flow. The TiO_2_|Pt photocatalyst was dispersed in ethanol (20 mg mL^–1^) by probe sonication (10 min, pulses of 30 s at 100% amplitude followed by 5 s pauses) followed by the addition of 1 vol % Nafion solution (5 wt %) to the resultant mixture. The dispersion was carefully drop-casted onto clean frosted glass (total of 16 μL cm^–2^ at a time) and dried for 10 min before the addition of subsequent layers (a total of 6 layers were added; final catalyst loading of ∼1.92 mg_cat_ cm^–2^). The prepared TiO_2_|Pt sheets were then annealed at 80 °C overnight in air.

The TiO_2_|Pt photocatalyst sheets (effective area 3.5 × 3.5 cm^2^) were mounted on a custom-made, air-tight PEEK reactor equipped with a quartz window (Figures 2c and S10). 12 mL of the carbonate buffer with LCC enzyme (concentration: 1 μM) and a piece of transparent PET film (weight ∼240 mg) was added to the reactor and then properly sealed. The solution was purged with N_2_ (with 2% CH_4_ as an internal standard) and the reactor was then placed in a calibrated Newport Oriel solar simulator (AM 1.5G, 100 mW cm^–2^). The steady-state temperature inside the reactor was measured to be ∼33 °C and the solution was not stirred during the experiment. Aliquots of the solution were taken at regular time intervals for estimating the hydrolysis of PET using HPLC-UV and the gas from the headspace (50 μL) was analyzed for H_2_ evolution using GC (discussed below). The control experiment was carried out in pure blank buffer.

### Photoreforming of PET for CO_2_ Reduction

Prior to the photocatalysis experiments, in order to remove TPA from the LCC pretreated PET solution, the solution was acidified with 1 M HCl to a pH of 3, which led to the precipitation of TPA as a white precipitate (Figure S13f). The suspension was subsequently filtered using a syringe filter (0.2 mm) to obtain a clear solution. The clear solution was then subsequently neutralized with 1 M NaOH to the original pH of 6.5. Tests were also conducted directly with the enzyme pretreated solution without TPA removal.

In a glass photoreactor, 5 mg TiO_2_ was suspended in 1 mL of the aqueous enzyme pretreated PET solution before or after TPA precipitation (see above) followed by 2 mL of MeCN. A known amount (25 or 50 nmol) of the molecular catalyst **CotpyP** (from a freshly prepared 2 mM solution in H_2_O; 0.0125 mL for 25 nmol or 0.025 mL for 50 nmol **CotpyP**) was added while stirring.^24^ The photoreactor (∼3 mL solution) was capped with a rubber septum and purged with CO_2_ containing 2% CH_4_ as an internal gas chromatography standard for 15 min, followed by stirring for 15 min in the dark. The photoreactor (kept at 25 °C and stirred at 600 rpm) was then irradiated with simulated solar irradiation (AM 1.5G, 100 mW cm^–2^) equipped with a water filter to remove infrared radiation. The photocatalytic process was monitored periodically by sampling the headspace (typically after 24 and 48 h) by GC to monitor H_2_ and CO formation. Products in the solution (formate) were detected by ^1^H NMR spectroscopy in D_2_O (1:1 v/v photocatalysis solution/D_2_O). Control exclusion experiments were performed by omitting components of the photocatalytic system (TiO_2_, **CotpyP**, light, and CO_2_) at a time. For the photocatalytic control experiments in the absence of CO_2_, the photoreactor was purged with N_2_ containing 2% CH_4_ as an internal standard (15 min). Reference experiments directly using the electron donor were performed by using aqueous stock solutions containing 60 mM EG or 60 mM EG + TPA as the aqueous phase. The turnover numbers (TON) were calculated based on **CotpyP** assuming that all cobalt sites are active catalytic sites.

### Reversed-Phase HPLC for Monomer Quantification

The enzymatic reactions were stopped by adding equal volumes of methanol with 0.5% (v/v) formic acid. After centrifugation (16,000 *xg*, 10 min), the supernatants were separated in a Nucleodur C18 analytical EC standard column (5 μm, 4 × 125 mm, Macherey-Nagel) on a 1260 Infinity II (Agilent) HPLC. To detect TPA, the mobile phase running at 1 mL min^–1^ consisted of buffer A (0.1% formic acid in distilled water), buffer B (distilled water), and buffer C (acetonitrile). The infusion rate of buffer A was kept constant at 20%; those of B and C was altered (5 min, 72% and 8%; 13 min, 50% and 30%; 17 min, 30% and 50%; and 18 min, 10% and 70%). The monomers were detected at λ = 250 nm and quantified using calibration curves. To detect the release of HA, a new set of mobile phase mixtures of buffer A (Milli-Q water) and buffer B (acetonitrile) were used at a flow rate of 1 mL min^–1^. Buffer A was kept at 100% for 5 min. The mobile phase was changed gradually to 100% buffer B until 10 min. Afterward, it was changed back to 100% buffer A until 15 min. HA was detected at 190 and 220 nm by comparison with authentic samples and quantified by calibration curves.

### Photoreforming Product Detection and Quantification

The production of H_2_ and CO was detected by manual injection of gas from the reactor headspace (50 μL) into a Shimadzu GC-2010 Plus GC and quantified using CH_4_ as an internal standard.^22^ The oxidation products in the solution post-photoreforming were detected and quantified using ^1^H NMR using maleic acid as an internal standard. The CO_2_ and hydrocarbons were detected using an Agilent 7890A GC equipped with a flame ionization detector (FID) and thermal conductivity detector (TCD).

### EQY Determination

The EQY measurements were carried out using a solar light simulator (LOT LSN 254) equipped with a monochromator (LOT MSH 300). The samples were irradiated with a wavelength of λ = 360 nm (for TiO_2_|Pt) and λ = 400 nm (for CN_*x*_|Ni_2_P). The light intensities were determined before and after each measurement and the irradiation area was kept fixed at 1 cm^2^. The EQY for the samples were determined using following eq 1
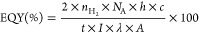
1where *n*_H_2__ is the amount of H_2_ formed (in mol), *N*_A_ is the Avogadro’s number (6.022 × 10^23^ mol^–1^), *h* is the Plank constant (6.626 × 10^–34^ m^2^ kg s^–1^), *c* is the velocity of light (3 × 10^8^ m s^–1^), *t* is the irradiation time (in s), *I* is the light intensity (in W m^–2^), λ is the wavelength (in m), and *A* is the irradiation area (in m^2^).

### Isotopic Labeling Experiments

To a glass photoreactor vial (7.7 mL total volume) equipped with a magnetic stir bar was added 5 mg of TiO_2_ suspended in 1 mL of aqueous stock solution (containing 60 mM EG, 60 mM TPA, 117 mM NaH^13^CO_3_, and 100 mM NaCl, pH 6.5) followed by 2 mL of MeCN. The molecular catalyst **CotpyP** (0.025 mL, 50 nmol, and 2 mM in H_2_O) was added and the photoreactor was capped with a rubber septum. The photoreactor was then degassed for 1 min (vacuum at 10^–2^ mbar) after which ^12^CO_2_ or ^13^CO_2_ (1 bar) was introduced. The photoreactor (kept at 25 °C and stirred at 600 rpm) was then irradiated (AM 1.5G, 100 mW cm^–2^) for 48 h. The headspace was transferred to an air-tight evacuated IR cell (10 cm path length, equipped with KBr windows) and the background (IR cell under vacuum) corrected IR spectrum was recorded to detect ^12^CO and ^13^CO. Isotopic labeling experiments were also performed with the stock solution followed by the TPA precipitation protocol before and after neutralization with 1 M NaOH (see the Photoreforming of PET for CO2 Reduction section).

### Metrics and Treatment of Data

The measurements are represented as yields of gas (H_2_ or CO): μmol per weight of the substrate (μmol g_sub_^–1^) and activity: μmol per weight of the photocatalyst per hour (μmol g_cat_^–1^ h^–1^). For the experiments with the integrated system, the H_2_ evolution data are represented in terms of the aerial efficiency of the photocatalyst sheet (μmol m_irr_^–2^). Unless otherwise indicated, the analytical measurements were performed in triplicates and represented as the unweighted mean ± standard deviation.

## Supporting Information Available

Monomer production from enzymatic pretreatment (MP4)
Enzyme pretreatment details; materials characterization; ^1^H NMR spectra; digital photographs of experimental setups; additional experimental details; and technoeconomic analysis (PDF)

## Notes

The raw data supporting the findings of this study are available from the University of Cambridge data repository: https://doi.org/10.17863/CAM.100189.
