# Supplementary material for: Chemoenzymatic Photoreforming: A Sustainable Approach for Solar Fuel Generation from Plastic Feedstocks
Source: J Am Chem Soc. 2023 Sep 6;145(37):20355–64. doi: 10.1021/jacs.3c05486 (PMC10515630; doi:10.1021/jacs.3c05486)
Supplement: Supplementary file 2 — ja3c05486_si_002.pdf [file ja3c05486_si_002.pdf]

Supporting Information

Chemoenzymatic Photoreforming: A Sustainable Approach for Solar Fuel Generation from Plastic Feedstocks

Subhajit Bhattacharjee,[1‡] Chengzhi Guo,[2‡] Erwin Lam,[1] Josephin M. Holstein,[2] Mariana Rangel Pereira,[2] Christian M. Pichler,[1] Chanon Pornrungroj,[1] Motiar Rahaman,[1] Taylor Uekert,[1] Florian Hollfelder[2,]* and Erwin Reisner[1,]*

‡ These authors contributed equally to this work

[1] *Yusuf Hamied Department of Chemistry, University of Cambridge, Lensfield Road, Cambridge, CB2 1EW, United Kingdom*
[2] *Department of Biochemistry, University of Cambridge, Cambridge, CB2 1GA, United Kingdom*

*** *Corresponding authors***
Prof. Florian Hollfelder; *Email*: fh111@cam.ac.uk
Prof. Erwin Reisner; *Email*: reisner@ch.cam.ac.uk

Supporting Figures

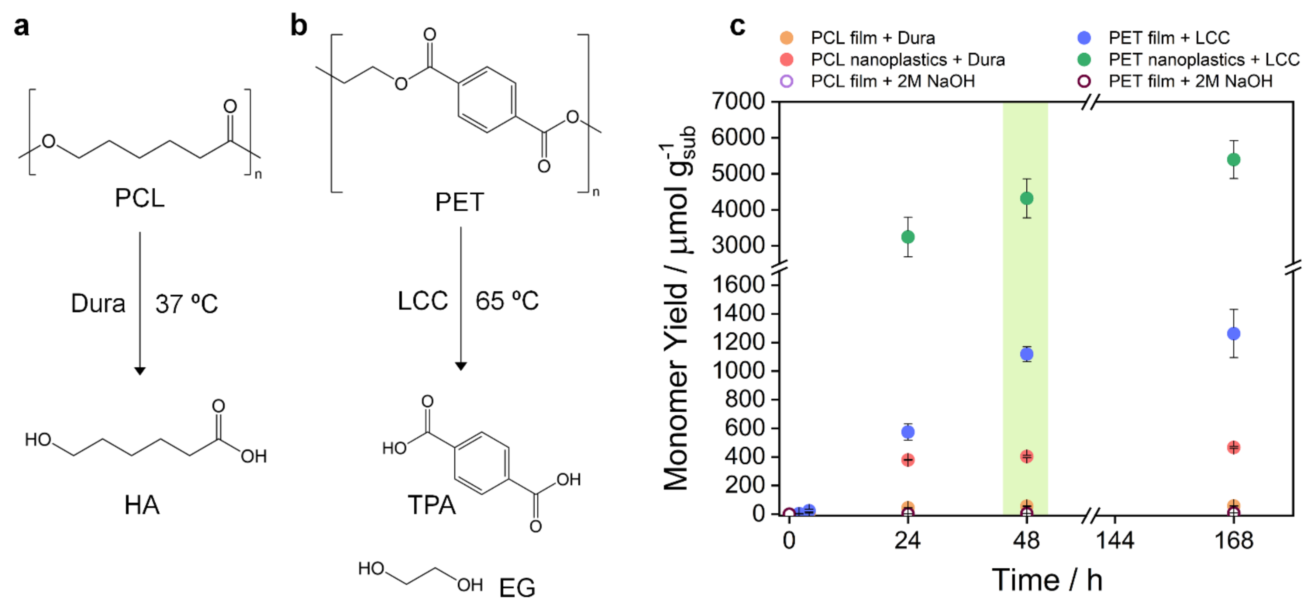

Figure S1. Monomer production from enzymatic pre-treatment. (a, b) Enzymatic depolymerization of PCL and PET amorphous polymers under the optimal temperatures of each enzyme, DuraPETase (37 °C)[1] and LCC (65 °C)[2], respectively in carbonate buffer (100 mM, pH 8.5). (c) Monomer yields from enzymatic pre-treatment traced using HPLC-UV (HA from PCL; TPA from PET). The samples were taken after two days (marked in green) for the photoreforming experiments. Alkaline pre-treatment with 2 M NaOH at identical temperatures to enzyme pre-treatment samples (*i.e.*, at 37 °C for PCL and 65 °C for PET films) yield sub-10 µM monomer amounts (open circles).

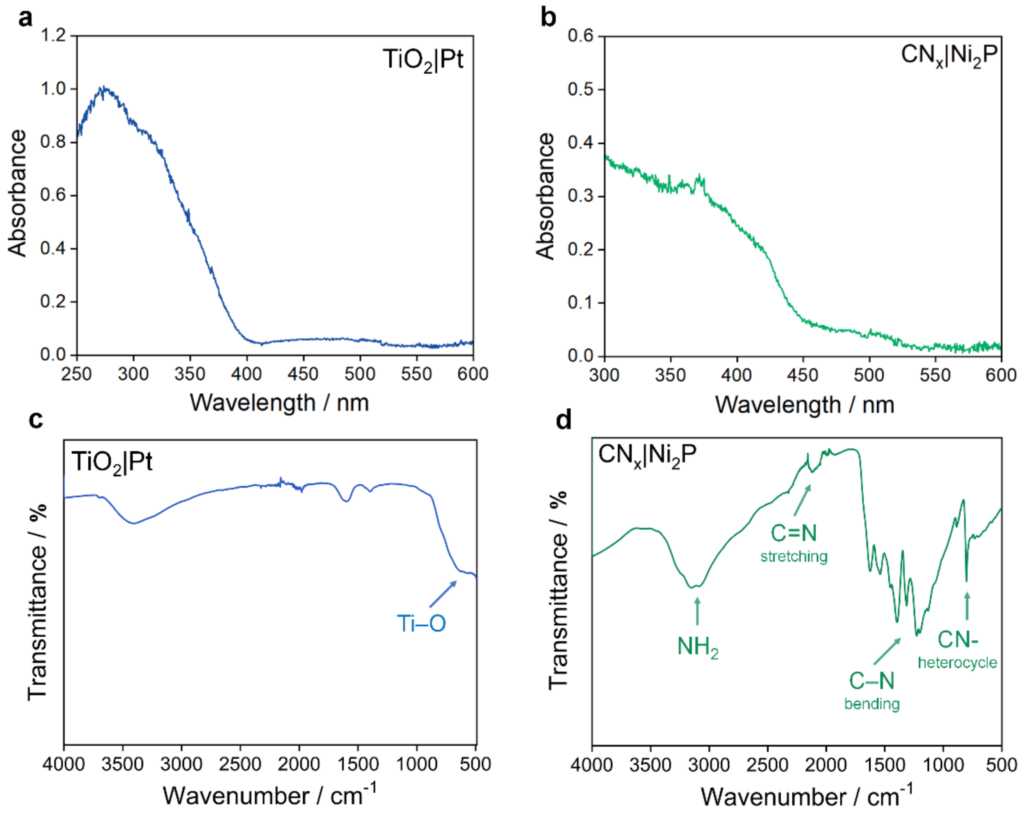

Figure S2. UV-vis and FT-IR spectra for the photocatalysts. (a, b) Solid-state UV-vis spectra of (a) TiO_2|Pt and (b) CN_x|Ni_2P photocatalysts. (c, d) FT-IR spectra of the (c) TiO_2|Pt and (d) CN_x|Ni_2P photocatalysts. While TiO_2|Pt absorbs strongly in the UV region, the precious metal-free CN_x|Ni_2P has an absorption onset around ~460 nm. The FT-IR of TiO_2|Pt shows Ti–O vibrational modes between 500–700 cm^{-1} while in the case of CN_x|Ni_2P, the vibrations appear at 804 cm^{-1} (corresponding to the heptazine core), between 1132 and 1411 cm^{-1} (–CN bending modes), and at ~ 2145 cm^{-1} (C=N stretch).

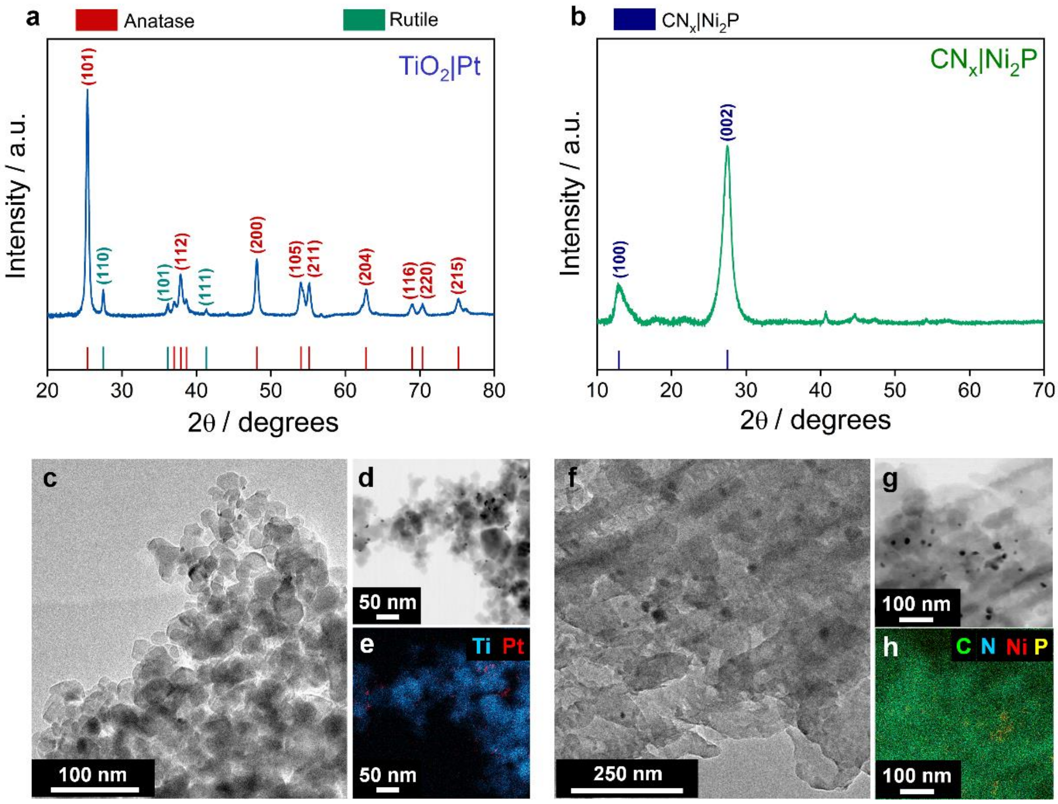

Figure S3. Structural characterization of the photocatalysts. (a, b) PXRD patterns of (a) TiO_2|Pt and (b) CN_x|Ni_2P photocatalysts. (c) TEM of TiO_2|Pt. (d) STEM-BF image and corresponding (e) elemental map of TiO_2|Pt. (f) TEM image of CN_x|Ni_2P. (g) STEM-BF image and corresponding (h) elemental map of CN_x|Ni_2P. The size of Pt particles ranged from ~4–16 nm on TiO_2, whereas that of Ni_2P ranged from ~4–15 nm on CN_x.

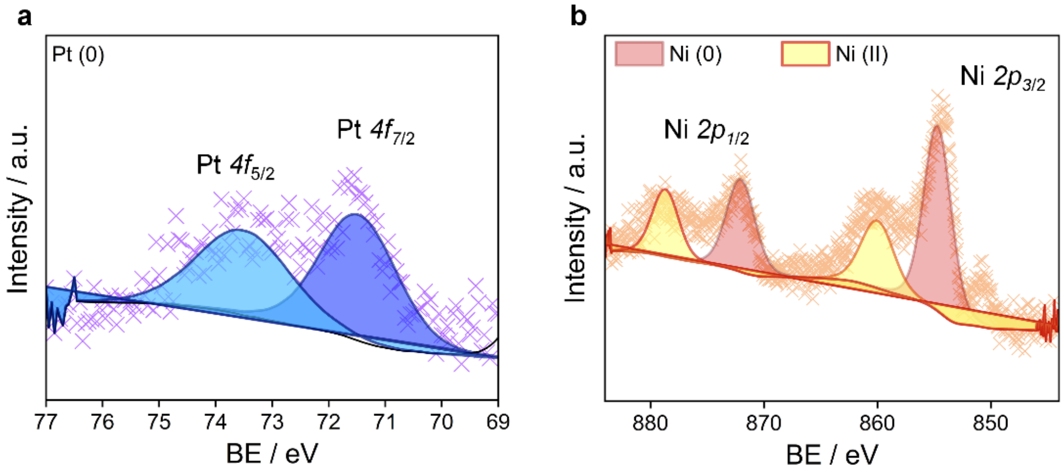

Figure S4. XPS spectra for co-catalysts. (a) Deconvoluted XPS spectra for TiO_2|Pt in the Pt *4f* region. (b) Deconvoluted XPS spectra for CN_x|Ni_2P in the Ni *2p* region. Traces of Ni (II) on the surface arises from NiO formed by surface aerial oxidation.

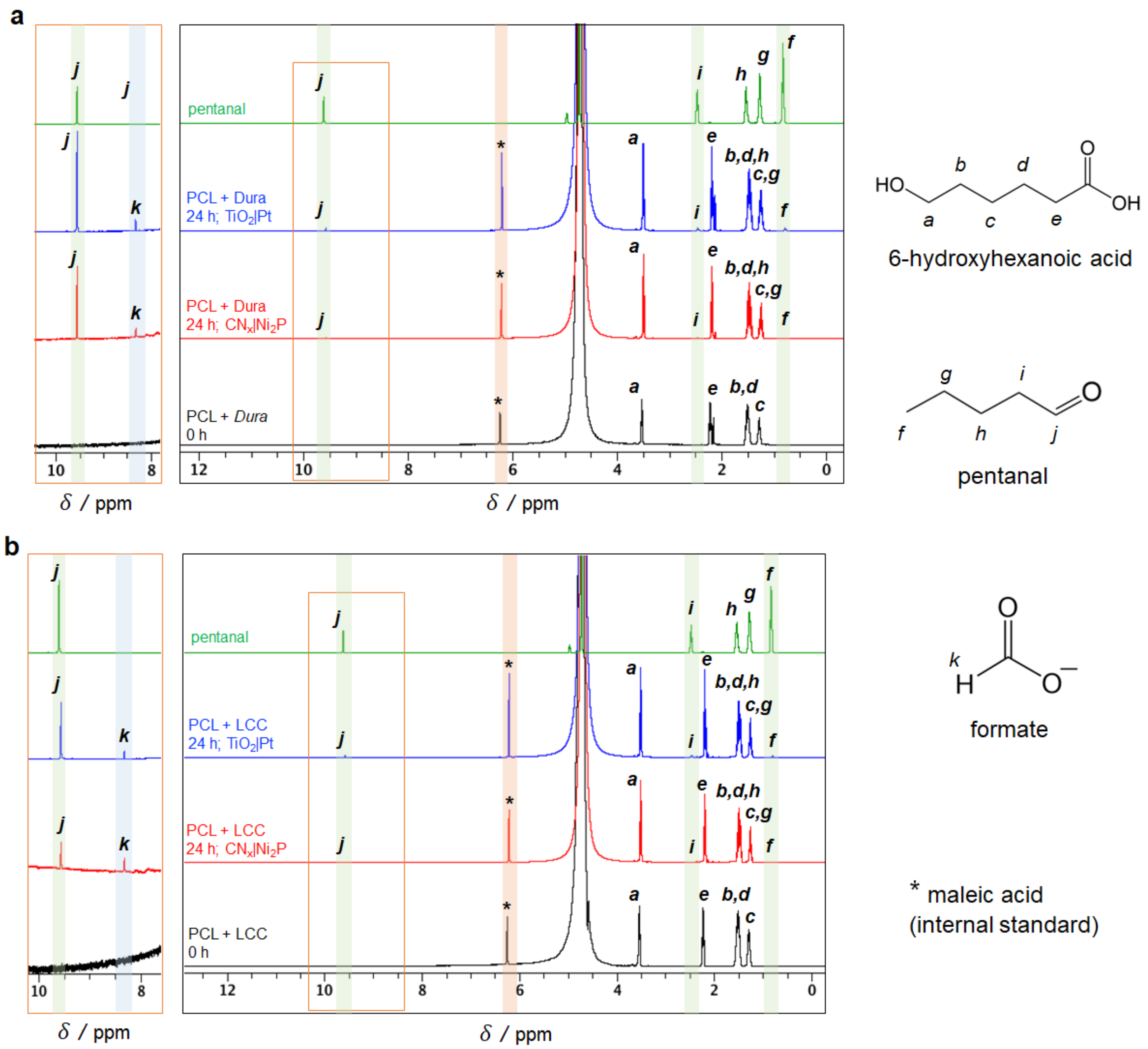

Figure S5. ^{1}H NMR analysis of the PCL films after photoreforming. (a) Stacked ^{1}H NMR spectra of Dura-treated PCL film before (black) and after photoreforming with $CN_x|Ni_2P$ (red) and $TiO_2|Pt$ (blue) photocatalysts. (b) Stacked ^{1}H NMR spectra of LCC-treated PCL film before (black) and after photoreforming with $CN_x|Ni_2P$ (red) and $TiO_2|Pt$ (blue) photocatalysts. Conditions: Photocatalyst concentration: 2 mg mL^{-1}; carbonate buffer (pH 6); AM 1.5G irradiation; 25 ºC; 24 hours; stirring. The panel on the left indicates the magnified region marked in the spectra. The pentanal reference ^{1}H NMR spectra is shown in green. The structures are shown in right with the corresponding marking of protons.

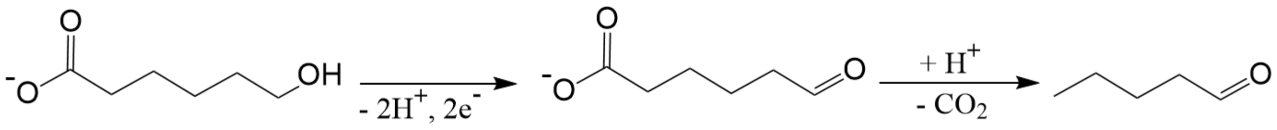

Figure S6. The oxidation schematic for the monomer 6-hydroxyhexanoic acid (derived from PCL plastic after enzymatic pre-treatment) during the photoreforming process.

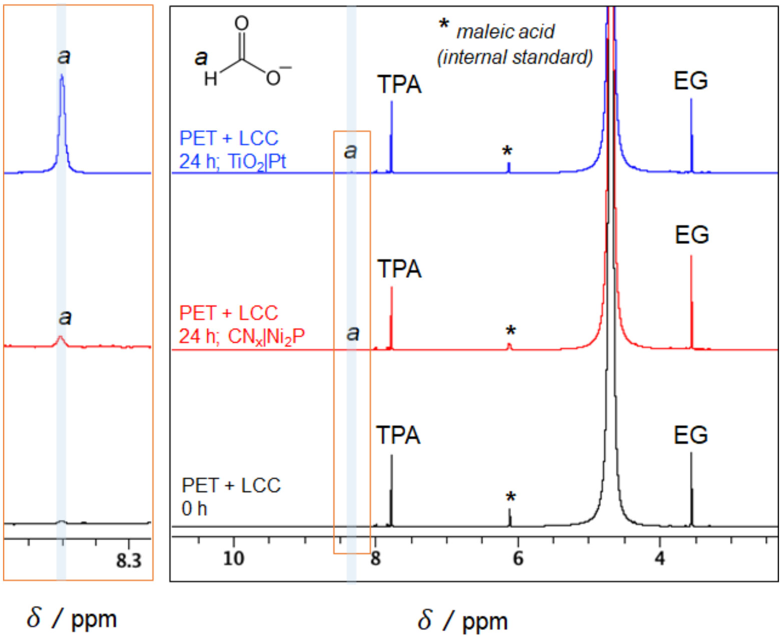

Figure S7. ^{1}H NMR analysis of the PET films after photoreforming. Stacked ^{1}H NMR spectra of LCC-treated PET film before (black) and after photoreforming with CN_x|Ni_2P (red) and TiO_2|Pt (blue) photocatalysts. Conditions: Photocatalyst concentration: 2 mg mL^{-1}; carbonate buffer (pH 6); AM1.5G irradiation; 25 ºC; 24 h; stirring. The panel on the left indicates the magnified region marked in the spectra. EG indicates 'ethylene glycol' and TPA indicates 'terephthalic acid'.

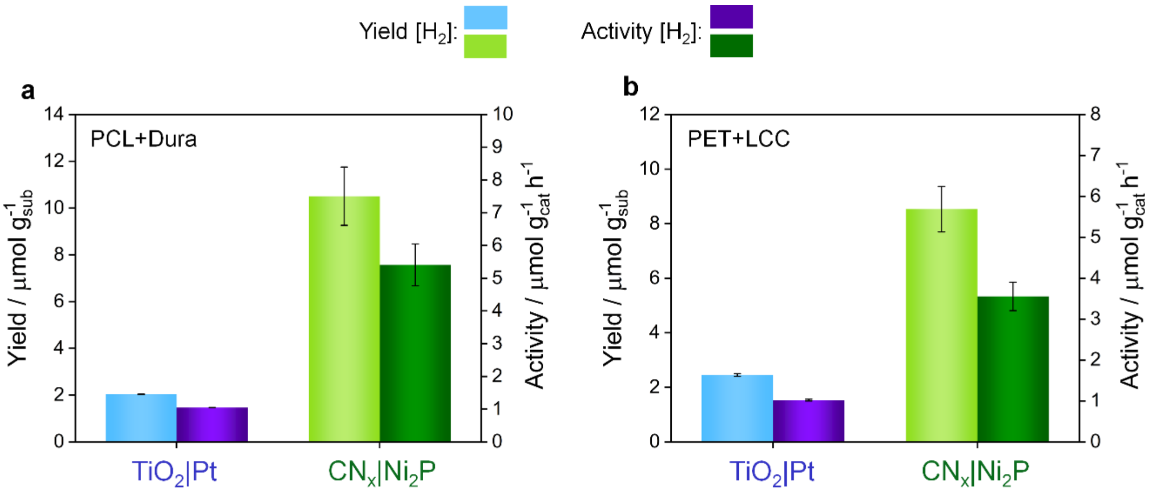

Figure S8. Photoreforming studies with visible light (λ>410 nm). (a, b) Bar plots showing the yield and activity of H_2 production from (a) Dura-treated PCL and (b) LCC-treated PET films employing TiO_2|Pt and CN_x|Ni_2P photocatalysts using λ>410 nm cut-off filter. Conditions: Photocatalyst concentration: 2 mg mL^{-1}; carbonate buffer (pH ~6); AM 1.5G irradiation; 25 ºC; 24 h; stirring.

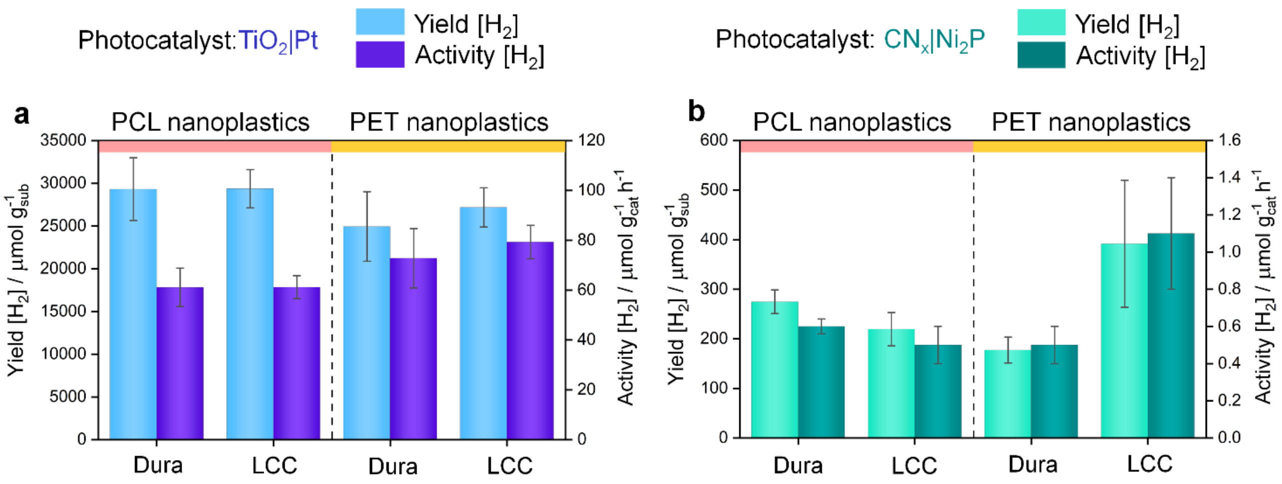

Figure S9. Photoreforming of enzyme-treated (enzyme: Dura or LCC) nanoplastics for H_2 evolution under benign conditions. (a, b) Bar plots showing the yield and activity of H_2 production from enzyme-treated PCL and PET nanoplastics employing (a) TiO_2|Pt and (b) CN_x|Ni_2P photocatalysts. Conditions: Photocatalyst concentration: 2 mg mL^{-1}; carbonate buffer (pH 6); AM 1.5G irradiation; 25 ºC; 24 h; stirring.

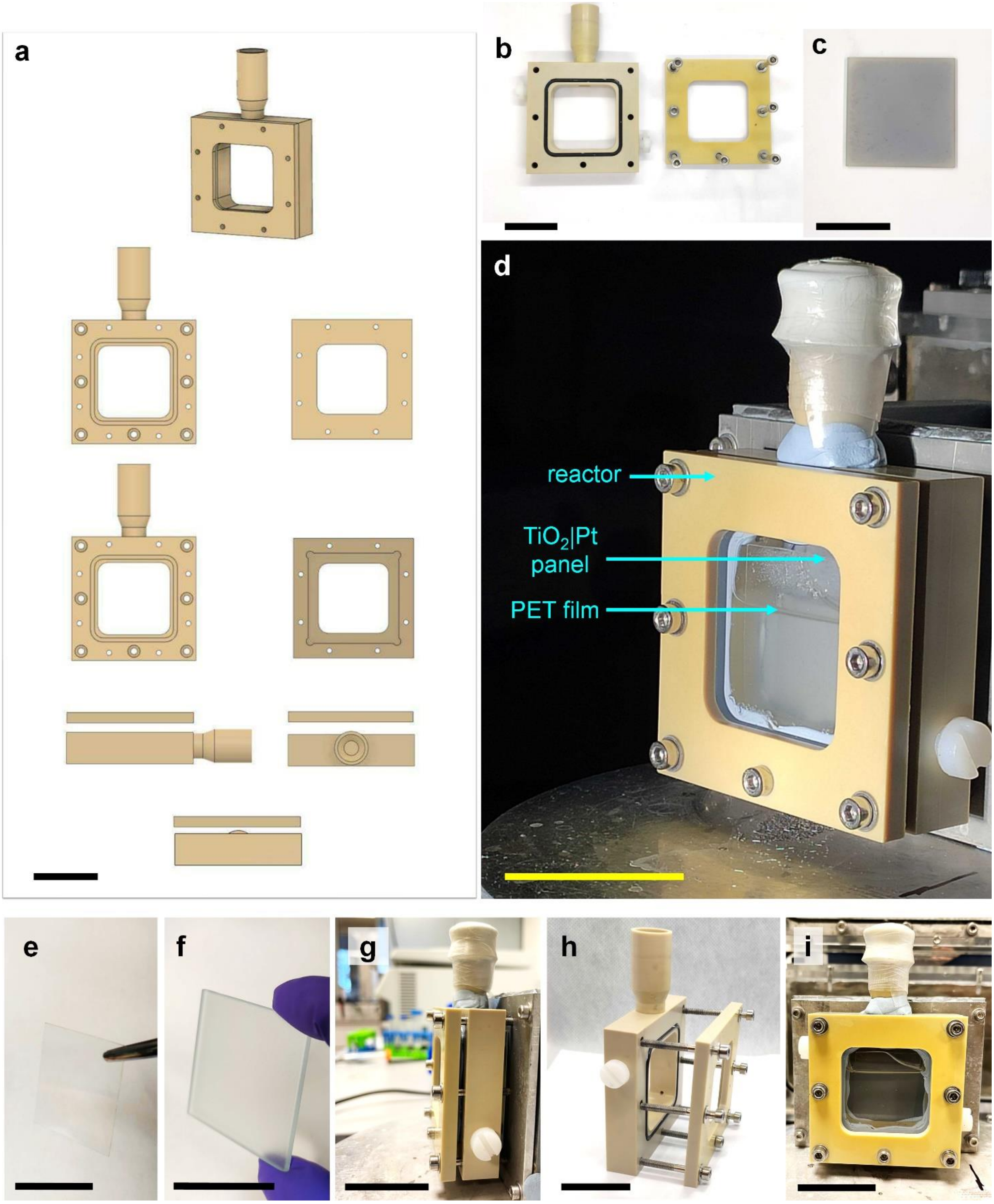

Figure S10. Assembly of the integrated system for photoreforming. (a) Graphics of the individual parts of the photoreactor. (b) Digital images of the frontal part of the photoreactor. (c) Digital image of a typical TiO_2|Pt panel. (d) A fully assembled reactor under operation. The PET film is placed in the buffer containing LCC. (e–i) Photographic images showing (e) the plastic film, (f) frosted glass substrate on which the catalyst is deposited as seen in (c), and (g–i) the reactor in three dimensions. The scale bars correspond to 3 cm.

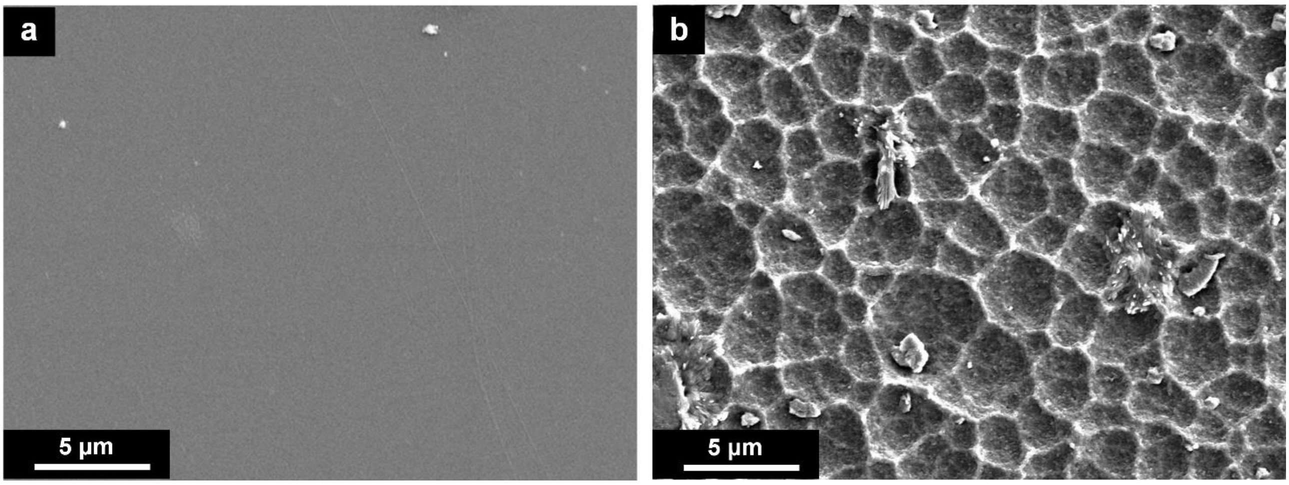

Figure S11. (a, b) FESEM images of the PET film (a) before and (a) after 96 h of enzymatic (LCC) pre-treatment inside the integrated system with simultaneous photoreforming. Before placing the film in the solution, it had an unstructured planar surface as shown in (a). However, a clear disfiguration of the PET film surface characterized by inhomogeneous craters caused by the LCC-enzyme was observed after 96 h as shown in (b).

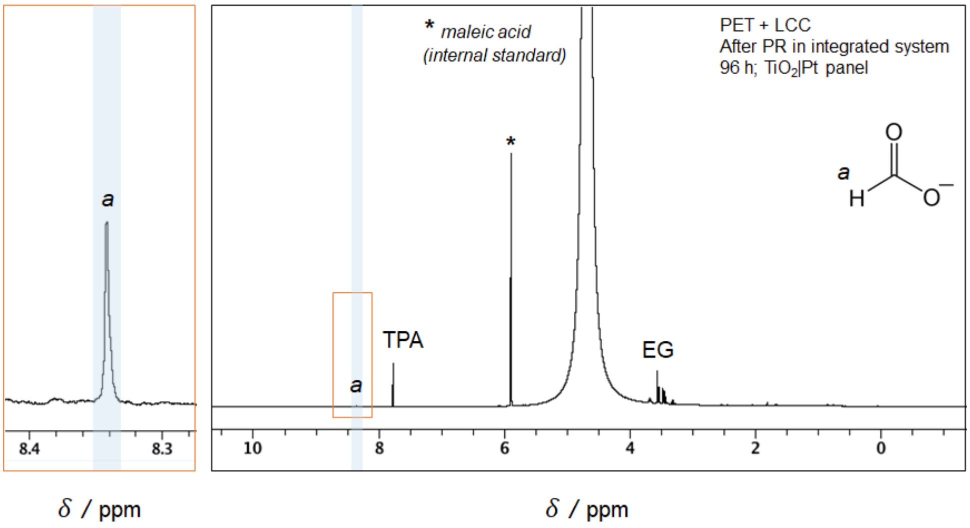

Figure S12. ^{1}H NMR analysis of the LCC-treated PET film after photoreforming in the integrated system. ^{1}H NMR spectra of LCC-treated PET film after photoreforming in the integrated system with a TiO_2|Pt panel (effective area 3.5 × 3.5 cm^2). Conditions: carbonate buffer (pH 6–8); AM 1.5G irradiation; 33 ºC; 96 h; stirring. The panel on the left indicates the magnified region marked in the spectra. EG indicates ‘ethylene glycol’ and TPA indicates ‘terephthalic acid’.

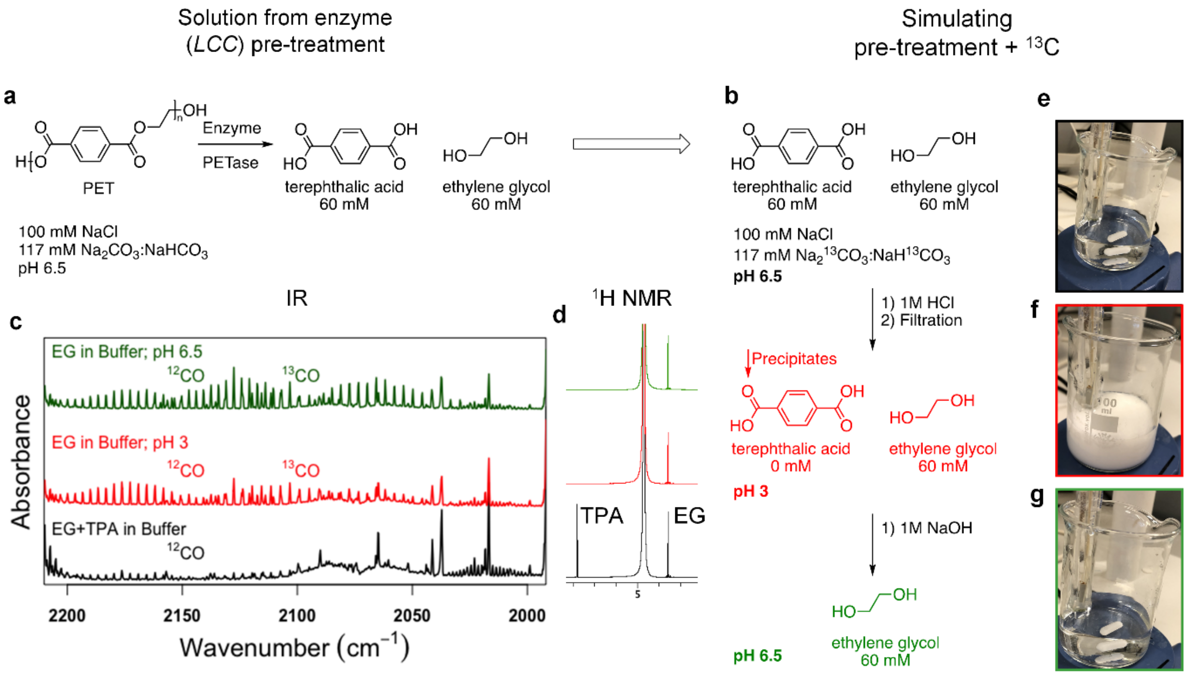

Figure S13. TPA removal protocol for photocatalytic CO_2 reduction experiments. (a) LCC-pre-treatment step and (b) steps for TPA removal from the pre-treated solution using simulated conditions. (c) The gas-phase IR spectra of the headspace after photocatalytic experiments with $^{13}CO_2$ purging at different stages (black: before TPA removal; red: TPA removal without neutralization; green: TPA removal + neutralization) of TPA removal process, showing the existence of both ^{13}CO and ^{12}CO. The presence of TPA (black) results in decreased activity (discussed in the main text) and inconclusive IR spectra. (d) The 1H-NMR of the solutions at different stages of TPA removal. (e-g) Photographs of the pre-treated solution at different stages of TPA removal: (e) without TPA removal, (f) TPA precipitation induced by acidic pH, and (g) filtered solution with pH-readjustment used for photocatalytic experiments.

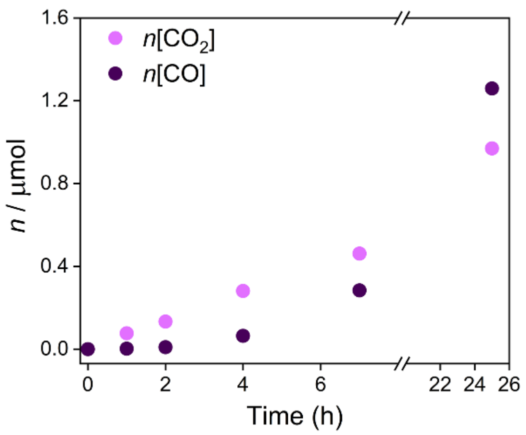

Figure S14. Time-dependent CO and CO_2 evolution. Amounts of CO and CO_2 evolved at various time intervals using 0.1 M EG substrate and TiO_2|**CotpyP** photocatalyst without external CO_2 purging. Conditions: MeCN:carbonate buffer with 0.1 M EG, N_2 purging, pH ~6.5; AM 1.5G irradiation; 25 ºC; stirring.

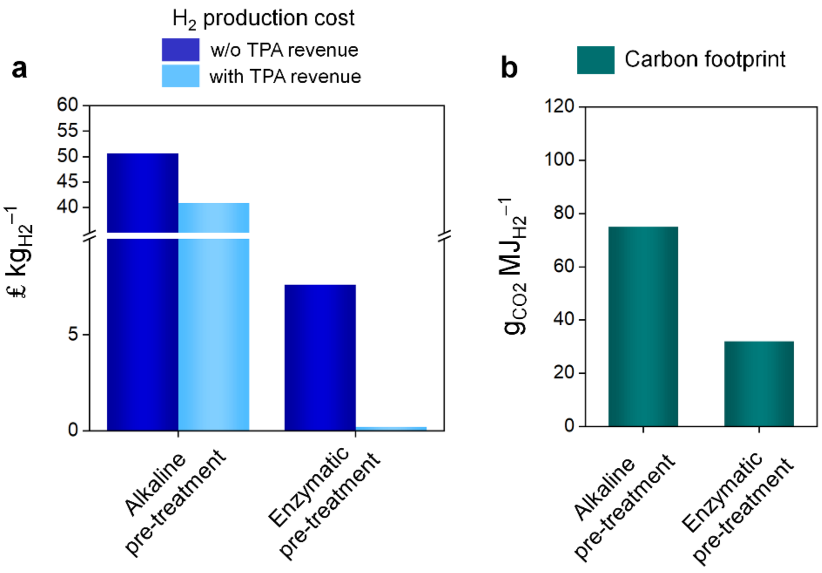

Figure S15. Techno-economic analyses results. Comparison of technoeconomic feasibility of alkaline pre-treatment and enzymatic pre-treatment processes for a 'base case' photoreforming pilot plant using the established metrics[3-4]: (a) cost of hydrogen production and (b) carbon footprint. The data for alkaline pre-treatment is adopted from previous reports.[3,5] See Supporting Discussion and Table S10 for details.

LCC

MNHHHHHHHHSGTSGDDASDRQTREVLDPIVASLMEAQQIPGMAIALVRPEGTTISHYGAADRETGTPVD
DDTLFEIGSLSKTLTATLASLAEVEGKLDFDAPVSRYLPELEGSAFDDISGLNLGTHTGGGLPLFVPDEVTD
RASLMAWYREWQPTEPIGESRTYSNLGIGLLGLETAASLDGEFVPTMRAKVLAPLGMQDTWYDVPEARM
ADYAMGEDKDGQPTRVSPGVLDDEAYGIKTTAADLAKLVRANLHLADVDAELQQAIDATRQGHYRVGDM
TQALIWEQYSLPVAPETLRAGNGYDMILEPNAAEALEPPQSPRDDVWVNKTGSTNGFGGYIVMLPGKHTG
LVMLANKNYPNDARVEAAYRILSGLGAIDVPSGTENLYFQGSNPYQRGPNPTRSALTADGPFSVATYTVSR
LSVSGFGGGVIYYPTGTSLTFGGIAMSPGYTADASSLAWLGRRLASHGFVVLVINTNSRFDYPDSRASQLSA
ALNYLRTSSPSAVRARLDANRLAVAGHSMGGGGTLRIAEQNPSLKAAVPLTPWHTDKTFNTSVPVLIVGAEA
DTVAPVSQHAIPFYQNLPSTTPKVYVELDNASHFAPNSNNAAISVYTISWMKLWVDNDTRYRQFLCNVNDPA
LSDFRTNNRHCQ*

DuraPETase

MGNPYARGPNPTAASLEASAGPFTVRSFTVSRPSGYGAGTVYYPTNAGGTVGAIAIVPGYTARQSSIKWWG
PRLASHGFVVITIDTNSTFDYPSSRSSQQMAALRQVASLNGDSSSPIYGKVDTARMGVMGHSMGGGASLRS
AANNPSLKAAIPQAPWDSQTNFSSVTVPTLIFACENDSIAPVNSHALPIYDSMSRNAKQFLEINGGSHSCANS
GNSNQALIGKKGVAWMKRFMDNDTRYSTFACENPNSTAVSDFRTANCSGAGSWSHPQFEK*

Figure S16. The amino acid sequence of LCC-pExp-Bla and DuraPETase construct. LCC was cloned into pExp-Bla vector containing 8x histag (in pink) and β-lactamase as a fusion tag (in cyan). LCC was cloned downstream of a TEV site (in red). The full-length recombinant protein has a molecular weight of 69.9 kDa. Dura was cloned into pHAT5 vector containing Strep-tag (in pink).

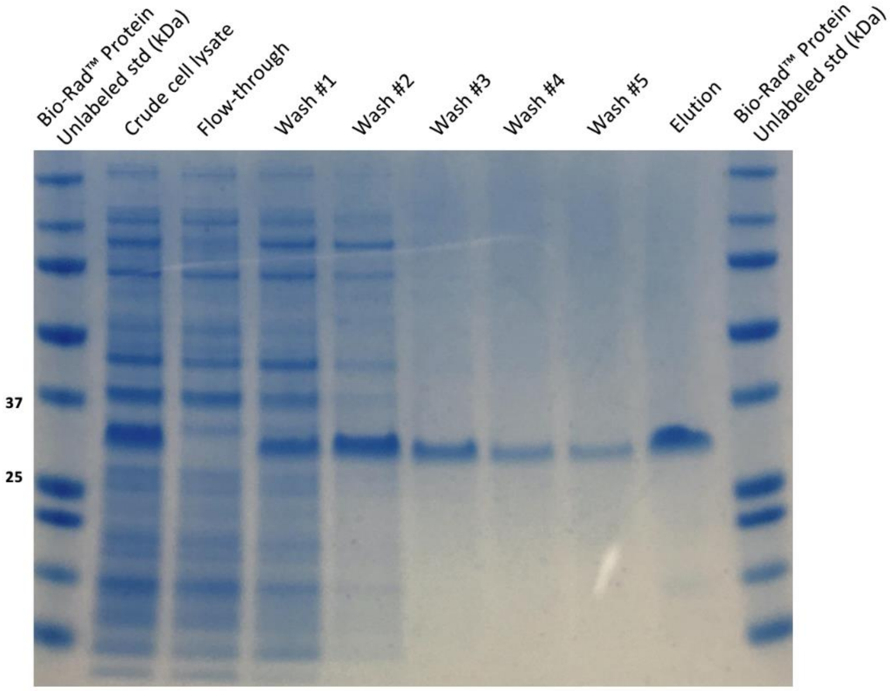

Figure S17. Expression and purification of DuraPETase construct. The recombinant protein DuraPETase was expressed overnight at 20 °C and the cells were lysed via emulsiflex. After centrifugation and removing cell debris, the DuraPETase (29 kDa) was purified using Strep-Tactin® Sepharose® resin according to the manufacturer's protocol.

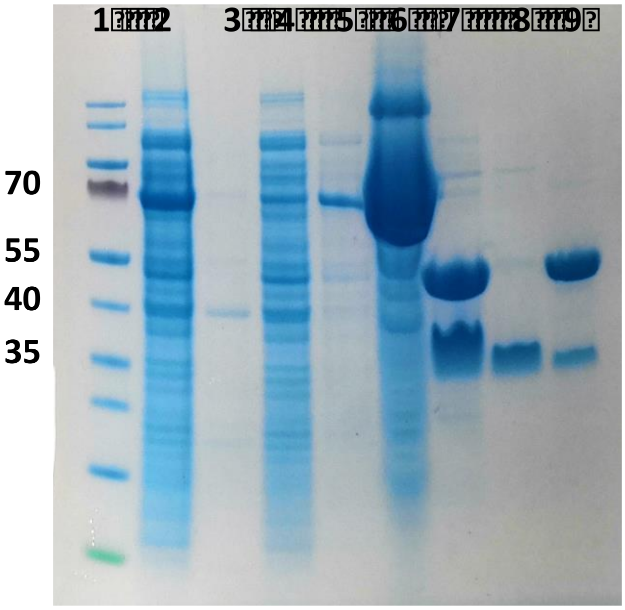

Figure S18. Expression, purification and TEV cleavage of LCC-pExp-Bla construct. The recombinant protein LCC-pExp-Bla was expressed overnight at 20 °C and the cells lysed via emulsiflex. After centrifugation and removing cell debris, the supernatant was loaded onto a column containing nickel resin for IMAC purification. To the fraction containing the purified LCC-pExp-Bla recombinant protein (6), TEV protease was added for cleavage overnight. The material was submitted to another IMAC purification step to obtain LCC without fusion tag (8). The numbers correspond to 1: protein ladder (Thermo Fisher Scientific, catalogue number 26616), 2: soluble fraction after protein extraction, 3: insoluble fraction, 4: flow-through, 5 and 6: purification fractions containing 10 and 200 mM of imidazole, respectively, 7: LCC after TEV cleavage overnight, 8: purified LCC, 9: Bla fusion tag and TEV protease.

Supporting Movies

Movie S1. Enzymatic degradation of PET film. The degradation/hydrolysis of a PET film is shown in the presence of LCC enzyme under benign conditions. Conditions: LCC enzyme (1 µM), 65 ºC, pH ~6–8, no stirring. The change in the shape and transparency of the PET film is visible with time suggesting enzymatic attack.

Supporting Discussion

Techno-economic Analysis

The benefit of the enzyme pre-treatment process for upscaled H_2 production and commercialisation is further realised with the help of techno-economic analyses. The base cases of previous reports inspire the model for our techno-economic analyses.[3-4] Both economic and environmental feasibility of the process was estimated using the metrics: H_2 production cost (£ kg_{H2}^{-1}), H_2 production cost with TPA revenue (£ kg_{H2}^{-1}), carbon footprint (g_{CO2} MJ_{H2}^{-1}) and energy returned on energy invested (EROI).[3]

We consider a base case chemoenzymatic photoreforming plant (plant lifetime assumed to be 20 years) to process 3 tons of PET plastic waste in 60000 L of solution per day with 180 kg TiO_2|Pt (1 wt% Pt loading) photocatalyst. This requires the plant to have 6000 m^2 of flat panel photoreactors with a 1 cm reactor depth.[3] A reactor depth of 1 cm was chosen to maximise the light-catalyst-substrate interaction.[3] Following pre-treatment overnight in a polypropylene tank, the solution is purged with N_2 (11250 L day^{-1}) and then pumped through the photoreactors. The solar intensity assumed is 1 Sun (100 mW cm^{-2}) for 7.5 h day^{-1}.[3] The H_2 produced is collected, compressed and stored in a tank (700 bar). The TPA is precipitated from the residual photoreforming mixture by adding sulfuric acid, filtered and crystallised as a secondary product to add economic value to the overall process.

Two cases are considered for our comparative techno-economic analyses: H_2 production from enzyme (LCC) pre-treated PET (our work) and a previously reported alkaline pre-treatment approach.[5] For the enzyme pre-treatment approach, sequential mechanical processing and overnight enzymatic pre-treatment are considered. According to previous reports,[4] PET depolymerisation efficiency was assumed to be ~69% (g_{TPA} g_{PET}^{-1}) overnight. The photocatalytic activities obtained experimentally (our work for enzymatic pre-treatment and previous report[5] for alkaline pre-treatment) were used to estimate the H_2 production, assuming a conversion rate of 50% (mol_{H2} mol_{sub}^{-1}) by the 'base case' models applied for techno-economic analyses in previous reports.[3] For the alkaline pre-treatment, the base (NaOH) is assumed to be reused 15 times.[3] The CO_2 production is considered five times the H_2 production for enzymatic pre-treatment, assuming that 50% of the CO_2 evolved is trapped as small organics as suggested in a previous report for near-neutral media.[3] For alkaline pre-treatment, no CO_2 is evolved as it is trapped as carbonate in the solution.[3] The transportation costs are not taken into consideration.[3]

The production costs, carbon footprint and EROI are calculated using the following equations:

<u>*Product production cost*</u>

$$Cost\ (£/kg) = \frac{C_{inv} + t \times (C_{op} + C_{con})}{t \times H}$$

Where, C_{inv} – capital investment cost (£); C_{op} – daily operational costs (£); C_{con} – daily consumables cost (£); H – daily H_2 production (kg); t – operation life of pilot plant (days).

<u>*Carbon footprint*</u>

$$Carbon\ Footprint\ (g\ CO_2 - eq/MJ\ H_2)\ =\ \frac{F_{inv} +\ t \times (F_{op} + F_{con} + F_{prod})}{t \times H}$$

Where, F_{inv} – carbon footprint of pilot plant construction and capital (g CO_2-eq); F_{op} – carbon footprint of daily operation (g CO_2-eq); F_{con} – carbon footprint of daily consumables (g CO_2-eq); F_{prod} – daily production of CO_2 from photoreforming (g); H – daily H_2 production (MJ, assuming lower energy density of 120 MJ kg^{-1}); t – operation life of plant (days).

EROI

$$EROI\ =\ \frac{t \times H}{E_{inv} +\ t \times (E_{op} + E_{con})}$$

Where, E_{inv} – embodied energy of pilot plant capital (MJ); E_{op} – daily operational energy (MJ); E_{con} – embodied energy of daily consumables (MJ); H – daily H_2 production (MJ, assuming lower energy density of 120 MJ kg^{-1}); t – operation life of plant (days).

The results obtained from our techno-economic analyses are shown in Figure S15 and listed in Tables S8-S10.

Major sources of deviations

For better comparison and uniformity, the technoeconomic calculations performed in this work assume a 'base case' model as discussed in previous reports.[3-4] Although detailed calculations and sensitivity analyses is beyond the scope of the current work, it is important to mention the major sources of deviations from the proposed model that may arise depending on the different parameters involved. Factors such as the choice of the (photo)catalyst material (and its efficiency), solar reforming configurations, enzymes utilised, feedstock scale, and pre-treatment method adopted will significantly alter the technoeconomic assessments. The deactivation of materials (e.g., enzymes, etc.), catalyst lifetime and duration of pilot plant operation will also play a vital role. Costs involving land acquisition, transportation, etc. which are not included in our simplified 'base case' also needs to be considered to provide a more holistic picture for future technoeconomic assessments. Other factors that may affect future estimates and commercial viability include sunlight intensities and geographical location of deployment, waste sourcing, competition, partnerships and customers identified, local and governmental regulations, carbon credits, etc. Therefore, all these factors need to be considered and evaluated to realize a viable solar-powered chemoenzymatic reforming plant.

Supporting Tables

Table S1. Monomer yields after enzyme pre-treated plastic substrates in duplicates. Conditions: carbonate buffer (100 mM, pH 8.5) at a total volume of 500 µL. Yields of 6-hydroxyhexanoic acid (HA; from pre-treated PCL) and terephthalic acid (TPA; from pre-treated PET) were measured using analytical HPLC-UV. Note: DuraPETase incubated at 65 °C with both PCL and PET films yielded no detectable monomers using HPLC. 'n.m.' indicates "not measured", 'n.q.' indicates "not quantifiable", 'σ' indicates "standard deviation".

**Substrate** [a]	**Enzyme (or pretreatment method)**	**Temp. (°C)**	**Molar Yield [HA or TPA] ± σ (µmol g_{sub}^{-1})**				
			**2 h**	**4 h**	**Day 1**	**Day 2**	**Day 7**
PCL film (24.7 mg mL^{-1})	**Dura**	**37**	**3.6 ± 0.2**	**7.9 ± 0.5**	**44.9 ± 0.9**	**56.1 ± 2.0**	**57.6 ± 1.7**
	LCC	65	n.m.	n.m.	4.8 ± 0.1	4.7 ± 0.1	6.0 ± 0.2
	2 M NaOH	37	n.m.	n.m.	n.q.	n.q.	n.q.
PET film (20 mg mL^{-1})	Dura	37	0.01 ± 0.0	0.01 ± 0.0	0.1 ± 0.01	2.04 ± 0.7	38.2 ± 8.1
	**LCC**	**65**	**3.6 ± 1.9**	**24.7 ± 9.1**	**575 ± 57**	**1120.0 ± 53**	**1260 ± 170**
	2 M NaOH	65	n.m.	n.m.	2.7 ± 0.3	4.8 ± 1.1	8.4 ± 0.3
PCL nanoplastics (~0.1 mg mL^{-1})	**Dura**	**37**	**n.m.**	**n.m.**	**379 ± 3.7**	**403 ± 7.4**	**466 ± 6.4**
PET nanoplastics (~0.1 mg mL^{-1})	**LCC**	**65**	**n.m.**	**n.m.**	**3240 ± 550**	**4320 ± 550**	**5390 ± 530**

[a] Substrates used are synthesised according to protocol detailed in the Methods section other than PET film, which was purchased from Goodfellow.

Table S2. Photoreforming of enzyme-treated PCL polymers (film and nano-plastics) in sealed photocatalytic vials (volume 7.91 mL). *Condition*: carbonate buffer (100 mM, pH ~6.0), AM 1.5G irradiation, 25 ºC, 24 h incubation. Abbreviations: OP indicates "oxidation product(s)", n.q. indicates "not quantifiable", resp. indicates "respectively", σ indicates "standard deviation".

Substrate	Enzyme	Photo-catalyst	$n[H_2] \pm \sigma$ (µmol)	Yield $[H_2] \pm \sigma$ ($\mu mol\ g_{sub}^{-1}$)	Activity $[H_2] \pm \sigma$ ($\mu mol\ g_{cat}^{-1}\ h^{-1}$)	OP	$n[OP] \pm \sigma$ (µmol)
PCL film (24.7 mg mL^{-1})	Dura	TiO_2|Pt (2 mg mL^{-1})	26.5 ± 2.1	1070 ± 85	553 ± 44	pentanal, formate, CO_2, hydrocarbons	4.8 ± 0.4, 0.5 ± 0.1, 5.1 ± 0.6, traces, resp.
PCL film (24.7 mg mL^{-1})	Dura	CN_x|Ni_2P (2 mg mL^{-1})	0.8 ± 0.1	32.1 ± 3.8	16.5 ± 2.0	pentanal, formate, CO_2, hydrocarbons	0.6 ± 0.4, <0.1, n.q., n.q., resp.
PCL film (24.7 mg mL^{-1})	LCC	TiO_2|Pt (2 mg mL^{-1})	20.1 ± 1.4	813 ± 56	418 ± 27	pentanal, formate, CO_2, hydrocarbons	2.5 ± 0.2, 0.5 ± 0.05, 2.6 ± 0.4, traces, resp.
PCL film (24.7 mg mL^{-1})	LCC	CN_x|Ni_2P (2 mg mL^{-1})	0.6 ± 0.2	22.4 ± 7.4	11.5 ± 3.8	pentanal, formate, CO_2, hydrocarbons	0.2 ± 0.05, <0.1, n.q., n.q., resp.
PCL nano-plastics (~ 0.1 mg mL^{-1})	Dura	TiO_2|Pt (2 mg mL^{-1})	2.9 ± 0.4	29300 ± 3700	61.1 ± 7.7	pentanal, formate, CO_2	traces
PCL nano-plastics (~ 0.1 mg mL^{-1})	Dura	CN_x|Ni_2P (2 mg mL^{-1})	0.03 ± 0.002	275 ± 24	0.6 ± 0.04	n.q.	n.q.
PCL nano-plastics (~ 0.1 mg mL^{-1})	LCC	TiO_2|Pt (2 mg mL^{-1})	2.9 ± 0.2	29400 ± 2200	61.2 ± 4.6	pentanal, formate, CO_2	traces
PCL nano-plastics (~ 0.1 mg mL^{-1})	LCC	CN_x|Ni_2P (2 mg mL^{-1})	0.02 ± 0.003	220. ± 34	0.5 ± 0.1	n.q.	n.q.
Controls							
-	Dura	TiO_2|Pt (2 mg mL^{-1})	1.1 ± 0.5	-	21.9 ± 9.4	-	-
-	Dura	CN_x|Ni_2P (2 mg mL^{-1})	0.013 ± 0.001	-	0.27 ± 0.01	-	-
-	LCC	TiO_2|Pt (2 mg mL^{-1})	2.5 ± 0.2	-	51.3 ± 4.3	-	-

-	LCC	$CN_x\|Ni_2P$ (2 mg mL^{-1})	0.03 ± 0.01	-	0.6 ± 0.3	-	-
PCL film (24.7 mg mL^{-1})	-	$TiO_2\|Pt$ (2 mg mL^{-1})	0.6 ± 0.3	24.9 ± 11.3	12.9 ± 5.8	-	-
PCL film (24.7 mg mL^{-1})	-	$CN_x\|Ni_2P$ (2 mg mL^{-1})	0.006 ± 0.002	0.2 ± 0.1	0.1 ± 0.04	-	-
PCL nano-plastics (~ 0.1 mg mL^{-1})	-	$TiO_2\|Pt$ (2 mg mL^{-1})	0.8 ± 0.1	7890 ± 1100	16.4 ± 2.3	-	-
PCL nano-plastics (~ 0.1 mg mL^{-1})	-	$CN_x\|Ni_2P$ (2 mg mL^{-1})	0.005 ± 0.001	45.5 ± 11.1	0.09 ± 0.02	-	-
PCL film (24.7 mg mL^{-1})	Dura	TiO_2 (2 mg mL^{-1})	0.2 ± 0.02	9.5 ± 0.9	4.8 ± 0.5	-	-
PCL film (24.7 mg mL^{-1})	Dura	CN_x (2 mg mL^{-1})	0.005 ± 0.001	0.2 ± 0.1	0.1 ± 0.03	-	-
PCL film (24.7 mg mL^{-1})	LCC	TiO_2 (2 mg mL^{-1})	0.3 ± 0.1	10.7 ± 2.4	5.5 ± 1.3	-	-
PCL film (24.7 mg mL^{-1})	LCC	CN_x (2 mg mL^{-1})	0.004 ± 0.001	0.1 ± 0.02	0.07 ± 0.01	-	-
PCL nano-plastics (~ 0.1 mg mL^{-1})	Dura	TiO_2 (2 mg mL^{-1})	0.003 ± 0.001	33.6 ± 11.6	0.07 ± 0.02	-	-
PCL nano-plastics (~ 0.1 mg mL^{-1})	Dura	CN_x (2 mg mL^{-1})	0.0	0.0	0.0	-	-
PCL nano-plastics (~ 0.1 mg mL^{-1})	LCC	TiO_2 (2 mg mL^{-1})	0.005 ± 0.002	51.6 ± 24.4	0.1 ± 0.05	-	-
PCL nano-plastics (~ 0.1 mg mL^{-1})	LCC	CN_x (2 mg mL^{-1})	0.0	0.0	0.0	-	-
-	-	$TiO_2\|Pt$ (2 mg mL^{-1})	0.6 ± 0.2	-	13.1 ± 3.8	-	-
-	-	$CN_x\|Ni_2P$ (2 mg mL^{-1})	0.006 ± 0.001	-	0.1 ± 0.02	-	-
PCL film (24.7 mg mL^{-1})	2M NaOH (37 ºC)	$TiO_2\|Pt$ (2 mg mL^{-1})	8.9 ± 1	449.1 ± 36.9	187.1 ± 15.4	-	-
PCL film (24.7 mg mL^{-1})	2M NaOH (65 ºC)	$TiO_2\|Pt$ (2 mg mL^{-1})	12.8 ± 0.4	641.3 ± 22.4	267.2 ± 9.3	-	-
Conditions with no catalyst and/or no light			0.0	0.0	0.0	-	-

Table S3. Photoreforming of enzyme-treated PET polymers (film and nano-plastics) under benign conditions in sealed photocatalytic vials. Conditions: carbonate buffer (100 mM, pH ~6), AM 1.5G irradiation, 25 ºC, 24 h. Abbreviations: OP indicates "oxidation product(s)", n.q. indicates "not quantifiable", resp. indicates "respectively", σ indicates "standard deviation".

Substrate	Enzyme	Photo-catalyst	$n[H_2] \pm \sigma$ (μmol)	Yield $[H_2] \pm \sigma$ ($\mu mol\ g_{sub}^{-1}$)	Activity $[H_2] \pm \sigma$ ($\mu mol\ g_{cat}^{-1}\ h^{-1}$)	OP	$n[OP] \pm \sigma$ (μmol)
PET film (20 mg mL^{-1})	Dura	TiO_2|Pt (2 mg mL^{-1})	1.3 ± 0.3	66.1 ± 16.6	27.5 ± 6.9	n.q.	n.q.
PET film (20 mg mL^{-1})	Dura	CN_x|Ni_2P (2 mg mL^{-1})	0.03 ± 0.01	1.5 ± 0.3	0.6 ± 0.1	n.q.	n.q.
PET film (20 mg mL^{-1})	LCC	TiO_2|Pt (2 mg mL^{-1})	24.9 ± 0.6	1240 ± 31	518 ± 13	formate, glycolate, oxalate	3.3 ± 0.2, n.q., n.q., resp.
PET film (20 mg mL^{-1})	LCC	CN_x|Ni_2P (2 mg mL^{-1})	0.5 ± 0.1	26.0 ± 5.2	10.8 ± 2.2	n.q.	n.q.
PET nano-plastics (~ 0.1 mg mL^{-1})	Dura	TiO_2|Pt (2 mg mL^{-1})	3.5 ± 0.6	24900 ± 4100	72.8 ± 11.9	n.q.	n.q.
PET nano-plastics (~ 0.1 mg mL^{-1})	Dura	CN_x|Ni_2P (2 mg mL^{-1})	0.02 ± 0.004	177 ± 26	0.5 ± 0.1	n.q.	n.q.
PET nano-plastics (~ 0.001 mg mL^{-1})	LCC	TiO_2|Pt (2 mg mL^{-1})	2.2 ± 0.4	2247930 ± 372868	46.8 ± 7.8	n.q.	n.q.
PET nano-plastics (~ 0.01 mg mL^{-1})	LCC	TiO_2|Pt (2 mg mL^{-1})	2.9 ± 0.6	298938 ± 61156	62.3 ± 12.7	n.q.	n.q.
PET nano-plastics (~ 0.1 mg mL^{-1})	LCC	TiO_2|Pt (2 mg mL^{-1})	3.8 ± 0.3	27200 ± 2300	79.3 ± 6.7	formate	traces
PET nano-plastics (~1 mg mL^{-1})	LCC	TiO_2|Pt (2 mg mL^{-1})	4.1 ± 1.1	4107 ± 1062	85.5 ± 22.1	formate	traces
PET nano-plastics (~ 0.001 mg mL^{-1})	LCC	CN_x|Ni_2P (2 mg mL^{-1})	0.03 ± 0.01	34020 ± 10714	0.7 ± 0.2	n.q.	n.q.

PET nano-plastics (~ 0.01 mg mL^{-1})	LCC	CN_x|Ni_2P (2 mg mL^{-1})	0.04 ± 0.004	3796 ± 360	0.8 ± 0.07	n.q.	n.q.
PET nano-plastics (~ 0.1 mg mL^{-1})	LCC	CN_x|Ni_2P (2 mg mL^{-1})	0.1 ± 0.05	392 ± 130	1.1 ± 0.3	n.q.	n.q.
PET nano-plastics (~1 mg mL^{-1})	LCC	CN_x|Ni_2P (2 mg mL^{-1})	0.1 ± 0.02	70 ± 19	1.4 ± 0.4	n.q.	n.q.
Controls							
-	Dura	TiO_2|Pt (2 mg mL^{-1})	1.1 ± 0.5	-	21.9 ± 9.4	-	-
-	Dura	CN_x|Ni_2P (2 mg mL^{-1})	0.013 ± 0.001	-	0.27 ± 0.01	-	-
-	LCC	TiO_2|Pt (2 mg mL^{-1})	2.5 ± 0.2	-	51.3 ± 4.3	-	-
-	LCC	CN_x|Ni_2P (2 mg mL^{-1})	0.03 ± 0.01	-	0.6 ± 0.3	-	-
PET film (20 mg mL^{-1})	-	TiO_2|Pt (2 mg mL^{-1})	0.7 ± 0.4	34.9 ± 18.0	14.5 ± 7.5	-	-
PET film (20 mg mL^{-1})	-	CN_x|Ni_2P (2 mg mL^{-1})	0.014 ± 0.007	0.7 ± 0.3	0.3 ± 0.1	-	-
PET nano-plastics (~ 0.1 mg mL^{-1})	-	TiO_2|Pt (2 mg mL^{-1})	0.8 ± 0.5	5750 ± 3600	16.7 ± 10.6	-	-
PET nano-plastics (~ 0.1 mg mL^{-1})	-	CN_x|Ni_2P (2 mg mL^{-1})	0.01 ± 0.002	71.9 ± 16.9	0.2 ± 0.05	-	-
PET film (20 mg mL^{-1})	Dura	TiO_2 (2 mg mL^{-1})	0.0	0.0	0.0	-	-
PET film (20 mg mL^{-1})	Dura	CN_x (2 mg mL^{-1})	0.0	0.0	0.0	-	-
PET film (20 mg mL^{-1})	LCC	TiO_2 (2 mg mL^{-1})	0.2 ± 0.01	10.8 ± 0.8	4.5 ± 0.3	-	-
PET film (20 mg mL^{-1})	LCC	CN_x (2 mg mL^{-1})	0.0	0.0	0.0	-	-
PET nano-plastics (~ 0.1 mg mL^{-1})	Dura	TiO_2 (2 mg mL^{-1})	0.005 ± 0.001	34.1 ± 5.1	0.1 ± 0.02	-	-

PET nano-plastics (~ 0.1 mg mL^{-1})	Dura	CN_x (2 mg mL^{-1})	0.0	0.0	0.0	-	-
PET nano-plastics (~ 0.1 mg mL^{-1})	LCC	TiO_2 (2 mg mL^{-1})	0.008 ± 0.001	54.1 ± 10.3	0.2 ± 0.03	-	-
PET nano-plastics (~ 0.1 mg mL^{-1})	LCC	CN_x (2 mg mL^{-1})	0.0	0.0	0.0	-	-
-	-	TiO_2|Pt (2 mg mL^{-1})	0.6 ± 0.2	-	13.1 ± 3.8	-	-
-	-	CN_x|Ni_2P (2 mg mL^{-1})	0.006 ± 0.001	-	0.1 ± 0.02	-	-
PET film (20 mg mL^{-1})	2M NaOH (37 ºC)	TiO_2|Pt (2 mg mL^{-1})	6.4 ± 0.8	322.4 ± 41.2	134.3 ± 17.2	-	-
PET film (20 mg mL^{-1})	2M NaOH (65 ºC)	TiO_2|Pt (2 mg mL^{-1})	10.5 ± 1.2	526.6 ± 60.2	219.4 ± 25.1	-	-
Conditions with no catalyst and/or no light			0.0	0.0	0.0	-	-

Table S4. EQY determination for photoreforming of enzyme-treated film substrates. Conditions: TiO_2|Pt or CN_x|Ni_2P photocatalyst (2 mg mL^{-1}) in a sealed quartz cuvette (with active irradiation area of 1 cm^2) with N_2 purging. Samples irradiated with monochromatic light (λ = 360 nm for TiO_2|Pt and λ = 400 nm for CN_x|Ni_2P, full width at half maximum: 5, intensities taken as average of intensities before and after each measurement); time interval for each experiment: 2 h; room temperature; stirring.

**System**	**Photocatalyst**	**Light Intensity (mW cm^{-2})**	**n[H_2] (µmol)**	**EQY (%)**
PCL film + Dura	TiO_2|Pt	16.4 ± 1.7	6.5 ± 0.7	3.6 ± 0.01
PET film + LCC	TiO_2|Pt	16.1 ± 0.9	5.5 ± 0.6	3.2 ± 0.5
PCL film + Dura	CN_x|Ni_2P	17.5 ± 1.2	0.04 ± 0.005	0.02 ± 0.001
PET film + LCC	CN_x|Ni_2P	17.1 ± 1.5	0.04 ± 0.004	0.02 ± 0.004

Table S5. Comparison of our work with other photoreforming processes reported with relevant polymeric substrates. Unless otherwise mentioned, the experiments were carried out at room temperature (25 ºC). AM 1.5G corresponds to an irradiation intensity of 1000 W m^{-2}. OP indicates "oxidation product(s)", n.m. indicates "not measured", resp. indicates "respectively", n.q. indicates "not quantifiable".

Sl. No.	Substrate	Pre-treatment conditions	Photocatalyst	Media	Irradiation	n[H_2] (µmol)	Yield (µmol g_{sub}^{-1})	Activity (µmol g_{cat}^{-1} h^{-1})	Oxidation products (OP)	n[OP] (µmol)	Ref.
**1**	PET powder (25 mg mL^{-1})	Strong alkaline (10 M NaOH, 40 ºC) , with stirring	TiO_2|Pt-5wt% (10 mg mL^{-1})	10 M aq. NaOH	AM 1.5G 20h	-	1220	153	n.m.	n.m.	5
**2**	PET powder (25 mg mL^{-1})	Strong alkaline (10 M NaOH, 40 ºC) , with stirring	CdS|CdO_x (0.1 mg mL^{-1})	10 M aq. NaOH	AM 1.5G 20h	-	460	4810	formate, glycolate, ethanol, acetate, lactate	n.m.	5
**3**	PET powder (25 mg mL^{-1})	Strong alkaline (2 M KOH, 40 ºC) , with stirring	$^{NCN}CN_x$|Ni_2P-2wt% (1.6 mg mL^{-1})	1 M aq. KOH	AM 1.5G 20h	-	33.1	25.8	acetate, formate, glycolate, glyoxal	~ 0.038, ~0.038, n.m., 1.8, resp.	6
**4**	PET microfibers (5 mg mL^{-1})	Strong alkaline (2 M KOH, 40 ºC) , with stirring	$^{NCN}CN_x$|Ni_2P-2wt% (1.6 mg mL^{-1})	1 M aq. KOH	AM 1.5G 24h	-	17.6	2.29	acetate, formate, glycolate, glyoxal	n.m.	6
**5**	PET powder (25 mg mL^{-1})	Strong alkaline (2 M KOH, 40 ºC) , with stirring	CN_x|Ni_2P-2wt% (1.6 mg mL^{-1})	1 M aq. KOH	AM 1.5G 20h	-	34.4	26.8	acetate, formate, glycolate, glyoxal	n.m.	6
**6**	PET powder (25 mg mL^{-1})	Strong alkaline (2 M KOH, 40 ºC) , with stirring	$^{NCN}CN_x$|Pt 2wt% (1.6 mg mL^{-1})	1 M aq. KOH	AM 1.5G 20h	-	96.2	75.2	acetate, formate, glycolate, glyoxal	n.m.	6
**7**	PET powder (25 mg mL^{-1})	Strong alkaline (2 M KOH, 40 ºC) , with stirring	TiO_2|Ni_2P-2wt% (1.6 mg mL^{-1})	1 M aq. KOH	AM 1.5G 20h	-	13.8	10.8	acetate, formate, glycolate, glyoxal	n.m.	6
**8**	PET powder (25 mg mL^{-1})	Strong alkaline (0.5 M KOH, 80 ºC) , with stirring	CN_x|Ni_2P panel (1.92 mg cm^{-2} ;1 cm^2)	0.5 M aq. KOH	AM 1.5G 20h	Areal efficiency: 3120 µmol m^{-2}	6.24	8.12	n.m.	n.m.	7

**9**	PET powder (25 mg mL^{-1})	Strong alkaline (0.5 M KOH, 80 ºC) , with stirring	$CN_x \vert Ni_2P$ panel (1.92 mg cm^{-2} ;25 cm^2)	0.5 M aq. KOH	AM 1.5G 12h[a] *flow setup*	Areal efficiency: 650 µmol m^{-2}	1.3	2.83	n.m.	n.m.	7
**10**	PCL film (24.7 mg mL^{-1})	Benign neutral Enzyme: **Dura** (~ 1 mg) pH 6, 37 ºC	$TiO_2 \vert Pt$-1wt% (2 mg mL^{-1})	Carbonate buffer (pH 6)	AM 1.5G 24h	26.5±2.1	1070±85	553±44	pentanal, formate, CO_2, hydrocarbons	4.8±0.4, 0.5±0.1, 5.1±0.6, traces, resp.	This work
**11**	PCL film (24.7 mg mL^{-1})	Benign neutral Enzyme: **LCC** (~ 1 mg) pH 6, 65 ºC	$TiO_2 \vert Pt$-1wt% (2 mg mL^{-1})	Carbonate buffer (pH 6)	AM 1.5G 24h	20.1±1.4	812±56	418±29	pentanal, formate, CO_2, hydrocarbons	2.5±0.2, 0.5±0.05, 2.6±0.4, traces, resp.	This work
**12**	PCL film (24.7 mg mL^{-1})	Benign neutral Enzyme: **Dura** (~ 1 mg) pH 6, 37 ºC	$CN_x \vert Ni_2P$-2wt% (2 mg mL^{-1})	Carbonate buffer (pH 6)	AM 1.5G 24h	0.8±0.1	32.1±3.8	16.5±2.0	pentanal, formate, CO_2, hydrocarbons	0.6±0.4, <0.1, n.q., n.q., resp.	This work
**13**	PCL film (24.7 mg mL^{-1})	Benign neutral Enzyme: **LCC** (~ 1 mg) pH 6, 65 ºC	$CN_x \vert Ni_2P$-2wt% (2 mg mL^{-1})	Carbonate buffer (pH 6)	AM 1.5G 24h	0.6±0.2	22.4±7.4	11.5±3.8	pentanal, formate, CO_2, hydrocarbons	0.2±0.05, <0.1, n.q., n.q., resp.	This work
**14**	PET film (20 mg mL^{-1})	Benign neutral Enzyme: **Dura** (~ 1 mg) pH 6, 37 ºC	$TiO_2 \vert Pt$-1wt% (2 mg mL^{-1})	Carbonate buffer (pH 6)	AM 1.5G 24h	1.3±0.3	66.1±16.6	27.5±6.9	n.q.	n.q.	This work
**15**	PET film (20 mg mL^{-1})	Benign neutral Enzyme: **LCC** (~ 1 mg) pH 6, 65 ºC	$TiO_2 \vert Pt$-1wt% (2 mg mL^{-1})	Carbonate buffer (pH 6)	AM 1.5G 24h	24.8±0.6	1240±31	518±13	formate, glycolate, oxalate	3.3±0.2, n.q., n.q., resp.	This work
**16**	PET film (20 mg mL^{-1})	Benign neutral Enzyme: **Dura** (~ 1 mg) pH 6, 37 ºC	$CN_x \vert Ni_2P$-2wt% (2 mg mL^{-1})	Carbonate buffer (pH 6)	AM 1.5G 24h	0.03±0.01	1.5±0.3	0.6±0.1	n.q.	n.q.	This work

**17**	PET film (20 mg mL^{-1})	Benign neutral Enzyme: **LCC** (~ 1 mg) pH 6, 65 ºC	CN_x|Ni_2P-2wt% (2 mg mL^{-1})	Carbonate buffer (pH 6)	AM 1.5G 24h	0.5±0.1	26.0±5.2	10.8±2.2	n.q.	n.q.	This work
**18**	PCL nano-plastics (~0.1 mg mL^{-1})	Benign neutral Enzyme: **Dura** (~ 1 mg) pH 6, 37 ºC	TiO_2|Pt-1wt% (2 mg mL^{-1})	Carbonate buffer (pH 6)	AM 1.5G 24h	2.9±0.4	29300 ±3700	61.1±7.7	pentanal, formate, CO_2,	traces	This work
**19**	PCL nano-plastics (~0.1 mg mL^{-1})	Benign neutral Enzyme: **LCC** (~ 1 mg) pH 6, 65 ºC	TiO_2|Pt-1wt% (2 mg mL^{-1})	Carbonate buffer (pH 6)	AM 1.5G 24h	2.9±0.2	29400 ±2200	61.2±4.6	pentanal, formate, CO_2,	traces	This work
**20**	PCL nano-plastics (~0.1 mg mL^{-1})	Benign neutral Enzyme: **Dura** (~ 1 mg) pH 6, 37 ºC	CN_x|Ni_2P-2wt% (2 mg mL^{-1})	Carbonate buffer (pH 6)	AM 1.5G 24h	0.03±0.002	275±24	0.6±0.04	n.q.	n.q.	This work
**21**	PCL nano-plastics (~0.1 mg mL^{-1})	Benign neutral Enzyme: **LCC** (~ 1 mg) pH 6, 65 ºC	CN_x|Ni_2P-2wt% (2 mg mL^{-1})	Carbonate buffer (pH 6)	AM 1.5G 24h	0.02±0.003	220.±34	0.5±0.1	n.q.	n.q.	This work
**22**	PET nano-plastics (~0.1 mg mL^{-1})	Benign neutral Enzyme: **Dura** (~ 1 mg) pH 6, 37 ºC	TiO_2|Pt-1wt% (2 mg mL^{-1})	Carbonate buffer (pH 6)	AM 1.5G 24h	3.5±0.6	24900 ±4070	72.8±11.9	formate	traces	This work
**23**	PET nano-plastics (~0.1 mg mL^{-1})	Benign neutral Enzyme: **LCC** (~ 1 mg) pH 6, 65 ºC	TiO_2|Pt-1wt% (2 mg mL^{-1})	Carbonate buffer (pH 6)	AM 1.5G 24h	3.8±0.3	27200 ±2300	79.3±6.7	formate	traces	This work

**24**	PET nano-plastics (~0.1 mg mL^{-1})	Benign neutral Enzyme: **Dura** (~ 1 mg) pH 6, 37 ºC	CN_x|Ni_2P-2wt% (2 mg mL^{-1})	Carbonate buffer (pH 6)	AM 1.5G 24h	0.02±0.004	177±26	0.5±0.1	n.q.	n.q.	This work
**25**	PET nano-plastics (~0.1 mg mL^{-1})	Benign neutral Enzyme: **LCC** (~ 1 mg) pH 6, 65 ºC	CN_x|Ni_2P-2wt% (2 mg mL^{-1})	Carbonate buffer (pH 6)	AM 1.5G 24h	0.1±0.05	392±130	1.1±0.3	n.q.	n.q.	This work
**26**	Integrated system; PET film (~20 mg mL^{-1})	*in-situ* Enzyme: **LCC** (1 μM) pH 6-8, 33 ºC	TiO_2|Pt panel (3.5 × 3.5 cm^2 effective area)	Carbonate buffer (pH 6-8)	AM 1.5G 96h	Areal efficiency: 9784 μmol m^{-2}	-	-	formate, glycolate, oxalate	4.9, n.q., n.q., resp.	This work

[a] residence time (in flow)

Table S6. Photoreforming of enzyme (LCC) pre-treated PET film (~20 mg mL^{-1}) in a custom-made integrated system with TiO_2|Pt panel (effective area 3.5 × 3.5 cm^2) under benign conditions. Conditions: carbonate buffer (pH ~6-8), AM 1.5G irradiation, 33 ºC. OP indicates "oxidation product(s)".

System	Time (h)	n[H_2] (µmol)	Aerial efficiency [H_2] (µmol m_{irr}^{-2})	OP	n[OP] (µmol)
PET film + LCC enzyme	2	0.05	40.0		
	4	0.09	75.7		
	24	1.6	1280		
	48	3.6	2940		
	72	11.3	9230		
	96	11.9	9780	formate, glycolate, oxalate	4.9, n.q., n.q., resp.
blank buffer	2	0.07	58.2		
	4	0.1	102		
	24	0.15	126		
	48	0.18	152		
	72	0.25	203		
	96	0.3	243	-	-

Table S7. Photocatalytic CO_2 reduction using LCC-treated PET film as substrate under benign conditions in sealed photocatalytic vials. Conditions: 5 mg TiO_2|**CotpyP** catalyst in 3 mL of 2:1 MeCN:(PET+LCC) solution purged with CO_2, AM 1.5G irradiation, 25 ºC. TON indicates "turn-over number", OP indicates "oxidation product(s)", n.q. indicates "not quantifiable", σ indicates "standard deviation". The pH of the enzyme pre-treated solutions was first decreased for TPA precipitation, followed by neutralization.

**Photo-catalyst**	**Substrate**	**Time (h)**	**n[CO] ± σ (μmol)**	**Yield [CO] ± σ (μmol g_{sub}^{-1})**	**Activity [CO] ± σ (μmol g_{cat}^{-1} h^{-1})**	**$n[H_2]$ ± σ (μmol)**	**Yield $[H_2]$ ± σ (μmol g_{sub}^{-1})**	**Activity $[H_2]$ ± σ (μmol g_{cat}^{-1} h^{-1})**	**TON [CO]**	**TON $[H_2]$**	**OP**	**n[OP] ± σ (μmol)**
TiO_2|**CotpyP** ***-10 nmol mg_{TiO2}^{-1}*** (1.67 mg mL^{-1})	pre-treated PET film (6.67 mg mL^{-1}) [***before*** *TPA removal + neutralization*]	24<br>48	0.17 ± 0.04<br>0.42 ± 0.09	8.5 ± 2<br>21 ± 5	1.4 ± 0.3<br>1.8 ± 0.4	1.63 ± 0.30<br>3.53 ± 0.68	81.5 ± 15<br>177 ± 34	13.6 ± 2.5<br>14.7 ± 2.8	3<br>8	33<br>71	formate	n.q.
TiO_2|**CotpyP** ***-10 nmol mg_{TiO2}^{-1}*** (1.67 mg mL^{-1})	pre-treated PET film (6.67 mg mL^{-1}) [***after*** *TPA removal + neutralization*]	24<br>48	0.81 ± 0.06<br>1.59 ± 0.12	40.5 ± 3<br>79.5 ± 6	6.8 ± 0.5<br>6.6 ± 0.5	3.58 ± 0.19<br>6.75 ± 0.34	179 ± 9.5<br>338 ± 17	29.8 ± 1.6<br>28.1 ± 1.4	16<br>32	72<br>135	formate	n.q.
TiO_2|**CotpyP** ***- 5 nmol mg_{TiO2}^{-1}*** (1.67 mg mL^{-1})	pre-treated PET film (6.67 mg mL^{-1}) [***after*** *TPA removal + neutralization*]	24<br>48	0.73 ± 0.01<br>1.39 ± 0.02	36.5 ± 0.5<br>69.5 ± 1	6.1 ± 0.1<br>5.8 ± 0.1	3.13 ± 0.13<br>5.56 ± 0.27	156.5 ± 7<br>278 ± 13.5	26.1 ± 1.1<br>23.2 ± 1.1	29<br>56	125<br>222	formate	n.q.
Controls												
TiO_2|**CotpyP** ***-10 nmol mg_{TiO2}^{-1}*** (1.67 mg mL^{-1})	pre-treated PET film (6.67 mg mL^{-1}) [***after*** *TPA removal* ***without neutralization***]	24	0.4 ± 0.09	20 ± 4.5	3.3 ± 0.8	1.83 ± 0.4	91.5 ± 20	15.3 ± 3.3	8	37	-	-

TiO_2|**CotpyP** ***-10 nmol mg$_{TiO2}$$^{-1}$*** (1.67 mg mL^{-1})	Pure monomers (EG+TPA)[a]	24<br>48	0.41 ± 0.06<br>0.89 ± 0.13	-	3.4 ± 0.5<br>3.7 ± 0.5	2.25 ± 0.4<br>4.54 ± 0.7	-	18.8 ± 3.3<br>18.9 ± 2.9	8<br>18	45<br>91	formate	-
TiO_2|**CotpyP** ***-10 nmol mg$_{TiO2}$$^{-1}$*** (1.67 mg mL^{-1})	only EG[a]	24	1.12 ± 0.2	-	9.3 ± 2	1.42 ± 0.1	-	11.8 ± 1	22	28	formate	-
TiO_2 (1.67 mg mL^{-1})	pre-treated PET film (6.67 mg mL^{-1})	24	0.1 ± 0.01	5 ± 0.5	0.8 ± 0.08	0.30 ± 0.15	15 ± 8	2.5 ± 1.3	2	6	-	-
TiO_2|**CotpyP** ***-10 nmol mg$_{TiO2}$$^{-1}$*** (1.67 mg mL^{-1})	pre-treated PET film (6.67 mg mL^{-1}) [***after*** *TPA removal + neutralization*] No CO_2 (N_2 purged)	24	0.35 ± 0.06	17.5 ± 3	2.9 ± 0.5	4.67 ± 0.44	234 ± 22	38.9 ± 3.6	7	93	formate	-
Conditions with no catalyst and/or no light		24	<0.01	-	-	<0.01	-	-	-	-	-	-

[a], *The same concentration of pure monomers was used as that obtained from the enzyme pre-treated samples.*

Table S8. Technoeconomic analyses for enzymatic pre-treatment. Values used for assessing photoreforming costs, carbon footprint and EROI, and individual component contributions to the ‘base case’ model[3-4] for our enzymatic pre-treatment process.

Component	Cost (£)	per unit	Ref.	Carbon Footprint (kg CO_2)	Per unit	Ref.	Embodied Energy (MJ)	Per unit	Ref.	Quantity	Cost (£)	Carbon Emissions (kg CO_2-eq)	Energy Embodied (MJ)
**Capital**													
Photocatalyst[a]	1030	kg		152.3	kg		2730	kg		180	185400	27414	491400
PVC cell chamber & assembly labor	52.7	m^2	13	17.6	m^2	11	533	m^2	13	6000	316200	105600	3198000
Plexiglass cover	30	m^2	14	3.2	m^2	11	66.9	m^2	13	6000	180000	19320	401400
Support frame	22.5	m^2	14	19	m^2	11	319	m^2	11	6000	135000	114000	1914000
PVC tubing	5	m	14	0.00036	m	11	0.009	m	11	6000	30000	2.16	54
Pretreatment container	1.7	L	15	0.25	L		0.943	L		60000	102000	15000	56580
Nylon filter to remove unreacted waste	0.77	m^2	15	0.009	m^2	16	0.287	m^2	17	150	115.5	1.365	43.05
Mechanical processing setup (converyor, extruder and microgranulator)	121065	unit	4	*Energy consumptions included in daily operation*						1	121065	-	-
TPA crystallization unit	268367	unit	4							1	268367	-	-
Pump	3000	unit								30	90000	-	-
H_2 compressor	40000	unit	18							15	600000	-	-
H_2 storage	2000	unit	19	20	kg	20	230	kg	21	45	90000	900	10350
Control systems	4.1	m^2	14	-			-			6000	24600	-	-
Installation labour	22.5	m^2	14	+30%		22	-			6000	135000	5096	-
Other system costs	43	m^2	14	-			-			6000	258000	-	-
Planning & consulting			23							+10%	208198	-	-
Administration & insurance			23							+2%	41639.6	-	-
Contingency planning			23							+15%	312297.1	-	-
Interest on investment										+10% p.a.	334545	-	-
Total (excluding TPA crystallization costs)											3164060	287334	6071827
**Daily Operation**													
Operating labour	15	h	24	-			-			4 h day^{-1}	60	-	-
Pretreatment power input	24.8	total	3	76	total	3	548	total	3	65 ºC/ 14 h	163.5	499.5	3607.5
Pump power (0.23 kW h^{-1})	0.037	h	3	0.044	h	3	0.083	h	3	82.8	0.89	1.06	1.99
H_2 compression (3.75 kWh kg H_2^{-1})	0.61	kg H_2	3	1.88	kg H_2	3	13.5	kg H_2	3	190.1	115.9	357.5	2566.8
Mechanical processing	-	-		0.6	kg_{PET}^{-1}	4	8.7	kg_{PET}^{-1}	4			1800	26100

TPA crystallization using H_2SO_4	0.028	kg_{PET}^{-1}	4	-	-		-	-		3000	85.4	-	-
Filtering using membranes	0.00014	kg_{PET}^{-1}	4	-	-		-	-		3000	0.42	-	-
Solid waste disposal	0.022	kg	25	0.015	kg	16	40	kg	26	300	6.6	4.41	12000
Water treatment	0.0016	L	27	0.0003	L	16	0.008	L	28	30000	46.5	9.6	240
Buffer & enzyme disposal	+5% (enzyme/buffer cost)					+5% operations					19.8	133.6	2225.8
Maintenance & repair				+ 5% of capital		23					433.4	-	-
Miscellaneous expenses				+ 5%							42.3	-	--
Total (excluding TPA crystallization costs)											889	2806	46742
Total (excluding TPA crystallization costs; operation over 20 year span)											6489996	20481060	341217521
**Daily Consumables**													
Water	0.0015	L	27	0.00032	L	16	0.008	L	28	30000	45	9.6	240
N_2	0.004	L	29	0.00054	L	16	0.003	L		11250	45	6.075	33.75
Biological buffers[b]	0.22	kg	27	0.79	kg	16	1.66	kg	30	900	198	711	1494
Enzyme expression and purification[c]	3.7	kg	31	0.325	kg	32	5.8	kg	32	0.00016	0.0006	0.000053	0.00094
Waste (municipal solid)[d]	-0.05	kg	33	0.29	kg	34	70	kg	26	3000	-159	0	0
Total											129	727	1768
Total (Consumables over 20 year span)											941704	5304728	12904582
Total (excluding TPA crystallization costs)											**10595760**	**26073122**	**360193930**
Total (including TPA crystallization costs)											**11490367**	**-**	**-**
**Daily Output**													
H_2										190 kg			22800
TPA										2070 kg			
CO_2	*~5 times of H_2 (50% trapped in solution)*									475 kg		475	
H_2 (kg; over 20 year span)										**1387984.5**			**166558137.4**
TPA (kg; over 20 year span)										**15111000**			
CO_2 (kg; over 20 year span)										**3469961.2**		**3499961.2**	
**TOTAL (without considering TPA revenue, carbon and energy offsets)**											**£ 7.6 kg_{H2}^{-1}**	**177.4 g_{CO2} MJ_{H2}^{-1}**	**0.5 MJ_{H2} MJ_{input}^{-1}**
**TOTAL (considering TPA: £ 0.74 kg_{TPA}^{-1}; 1.6 kgCO_2 kg_{TPA}^{-1}; 24 MJ kg_{TPA}^{-1})[4]**											**£ 0.22 kg_{H2}^{-1}**	**32.2 g_{CO2} MJ_{H2}^{-1}**	**1.5 MJ_{H2} MJ_{input}^{-1}**

[a] Calculated assuming a combination of TiO_2 (£30/kg, 5.3 kg CO_2-eq/kg, 30 MJ/kg)[8-10] and Pt nanoparticles (£100,000/kg, 14,700 kg CO_2-eq/kg, 270,000 MJ/kg).[11-12]
[b] Salt components for carbonate buffer (100 mM, pH 8.5).
[c] Costs of LCC-production was estimated from the industrial production of cellulases through fermentation (case 2).[30] Enzyme dosage level was estimated at 27 µg kg_{PET}^{-1}.
[d] The pilot plant would be paid a gate fee for taking plastic waste, hence the negative cost.[3]

Table S9. Technoeconomic analyses for alkaline pre-treatment. Values used for assessing photoreforming costs, carbon footprint, EROI, and individual component contributions for alkaline pre-treatment process based on base case of previous reports.[3-4] Only the components/parameters which have been newly added or modified from that in the case of enzymatic pre-treatment are shown in the table (rest remaining the same). The mechanical processing (required for greater enzyme activity), enzyme and buffer components are not considered for alkaline pre-treatment.

**Component**	**Cost (£)**	**per unit**	**Ref.**	**Carbon Footprint (kg CO_2)**	**Per unit**	**Ref.**	**Embodied Energy (MJ)**	**Per unit**	**Ref.**	**Quantity**	**Cost (£)**	**Carbon Emissions (kg CO_2-eq)**	**Energy Embodied (MJ)**
**Capital**													
Total (excluding TPA crystallization costs)											2992148	287333.5	6071827.1
**Daily Operation**													
Pre-treatment power input	24.8	total	3	76	total	3	548	total	3	40 ºC/ 14 h	100.5	307.5	2220
H_2 compression (3.75 kWh kg H_2^{-1})	0.61	kg H_2	3	1.88	kg H_2	3	13.5	kg H_2	3	55.9 kg	34.1	105	754
NaOH disposal	0.74	kg	35	0.46	kg	16	1.792	kg	36	1596 kg	1181	734.2	2860
Total (excluding TPA crystallization costs)											1931.5	1163.1	18076.1
Total (excluding TPA crystallization costs; operation over 20 year span)											14099646.7	8490871.8	131955173.9
**Daily Consumables**													
NaOH	0.35	kg	37	0.46	kg	16	1.792	kg	36	1596 kg	558.6	734.2	2860
Total											489.6	749.8	3133.8
Total (Consumables over 20 year span)											3574080	5473795.5	22876608.6
Total (excluding TPA crystallization costs)											**20665874.7**	**14252000.86**	**160903609.6**
Total (including TPA crystallization costs)											**21560481.5**	**-**	**-**
**Daily Output**													
H_2										55.9 kg			6708
TPA										906 kg			
CO_2										0 kg		0	
H_2 (kg; over 20 year span)										**407734.3**			**48928118.5**
TPA (kg; over 20 year span)										**6613800**			
CO_2 (kg; over 20 year span)										**0**		**0**	
**TOTAL (without considering TPA revenue, carbon and energy offsets)**											**£ 50.7 kg_{H2}^{-1}**	**291.3 g_{CO2} MJ_{H2}^{-1}**	**0.3 MJ_{H2} MJ_{input}^{-1}**
**TOTAL (considering TPA: £ 0.74 kg_{TPA}^{-1}; 1.6 $kgCO_2$ kg_{TPA}^{-1}; 24 MJ kg_{TPA}^{-1})[4]**											**£ 40.9 kg_{H2}^{-1}**	**75.0 g_{CO2} MJ_{H2}^{-1}**	**1.3 MJ_{H2} MJ_{input}^{-1}**

Table S10. Techno-economic analyses results and comparison. Summary of parameters/estimates for alkaline pre-treatment used for techno-economic calculations (data adopted from the previous report[5]) and estimates for our enzyme pre-treatment approach in accordance with base cases of previous reports with minor modifications.[3-4]

		Our base case		
**Variable**	**Unit**	**Alkaline pre-treatment[5]**	**Enzymatic pre-treatment**	**% change when adopting enzymatic pre-treatment**
Pre-treatment temperature	ºC	40	65 (for LCC)	
NaOH reuse	days	15	-	
Enzyme reuse	times (x)	-	no reuse	
Production cost of H_2 (20 year span, w/o TPA revenue)		**£ 50.7 kg_{H2}^{-1}**	**£7.6 kg_{H2}^{-1}**	**~85% reduction**
Production cost of H_2 (20 year span, with TPA revenue)		**£ 40.9 kg_{H2}^{-1}**	**£0.2 kg_{H2}^{-1}**	**~99.5% reduction**
Carbon footprint (20 year span without TPA offset)		**291 g_{CO2} MJ_{H2}^{-1}**	**177 g_{CO2} MJ_{H2}^{-1}**	**~39% reduction**
Carbon footprint (20 year span, considering TPA offset)		**75 g_{CO2} MJ_{H2}^{-1}**	**32 g_{CO2} MJ_{H2}^{-1}**	**~57% reduction**

Supporting References

(1) Cui, Y.; Chen, Y.; Liu, X.; Dong, S.; Tian, Y.; Qiao, Y.; Mitra, R.; Han, J.; Li, C.; Han, X.; Liu, W.; Chen, Q.; Wei, W.; Wang, X.; Du, W.; Tang, S.; Xiang, H.; Liu, H.; Liang, Y.; Houk, K. N.; Wu, B. Computational Redesign of a PETase for Plastic Biodegradation Under Ambient Condition by the GRAPE Strategy. *ACS Catal.* **2021**, *11*, 1340–1350.

(2) Tournier, V.; Topham, C. M.; Gilles, A.; David, B.; Folgoas, C.; Moya-Leclair, E.; Kamionka, E.; Desrousseaux, M.-L.; Texier, H.; Gavalda, S.; Cot, M.; Guémard, E.; Dalibey, M.; Nomme, J.; Cioci, G.; Barbe, S.; Chateau, M.; André, I.; Duquesne, S.; Marty, A. An Engineered PET Depolymerase to Break down and Recycle Plastic Bottles. *Nature* **2020**, *580*, 216–219.

(3) Uekert, T.; Pichler, C. M.; Schubert, T.; Reisner, E. Solar-driven Reforming of Solid Waste for a Sustainable Future. *Nat. Sustain.* **2021**, *4*, 383–391.

(4) Singh, A.; Rorrer, N. A.; Nicholson, S. R.; Erickson, E.; Desveaux, J. S.; Avelino, A. F. T.; Lamers, P.; Bhatt, A.; Zhang, Y.; Avery, G. *et al.* Techno-economic, Life-cycle, and Socioeconomic Impact Analysis of Enzymatic Recycling of Poly(ethylene Terephthalate). *Joule* **2021**, *5*, 2479–2503.

(5) Uekert, T.; Kuehnel, M. F.; Wakerley, D. W.; Reisner, E. Plastic Waste as a Feedstock For Solar-driven H_2 Generation. *Energ. Environ. Sci.* **2018**, *11*, 2853–2857.

(6) Uekert, T.; Kasap, H.; Reisner, E. Photoreforming of Non-recyclable Plastic Waste Over a Carbon Nitride/Nickel Phosphide Catalyst. *J. Am. Chem. Soc.* **2019**, *141*, 15201–15210.

(7) Uekert, T.; Bajada, M. A.; Schubert, T.; Pichler, C. M.; Reisner, E. Scalable Photocatalyst Panels for Photoreforming of Plastic, Biomass and Mixed Waste in Flow. *ChemSusChem* **2021**, *14*, 4190–4197.

(8) Kim, H. C.; Fthenakis, V. Life Cycle Energy and Climate Change Implication of Nanotechnologies. *J. Ind. Ecol.* **2013**, *17*, 528–541.

(9) Titanium Dioxide Manufacturers Association & European Chemical Industry Council. The Carbon Footprint of Titanium Dioxide Pigment, 2013.

(10) Nosaka, Y.; Nosaka, A. Introduction to Photocatalysis: From Basic Science to Applications. (The Royal Society of Chemistry, **2016**).

(11) Ashby, M. F. Materials and the Environment : Eco-informed Material Choice. (Butterworth Heinemann, **2012**).

(12) Platinum, 99.999%, (trace metal basis), powder, ACROS OrganicsTM 1g; Glass bottle Platinum Fisher Scientific.

(13) PVC Sheet | Direct Plastics Limited. Available at: https://www.directplastics.co.uk/pvc-sheet. (Accessed: 12 March 2022)

(14) Shaner, M. R.; Atwater, H. A.; Lewis, N. S.; Mcfarland, E. W. A Comparative Technoeconomic Analysis of Renewable Hydrogen Production Using Solar Energy. *Energy Environ. Sci.* **2016**, *9*, 2354–2371.

(15) Alibaba.com: Manufacturers, Suppliers, Exporters & Importers from the World's Largest Online B2B Marketplace. Available at: https://www.alibaba.com

(16) https://www.winnipeg.ca/finance/findata/matmgt/documents/2012/682-2012/682-2012_Appendix_H-WSTP_South_End_Plant_Process_Selection_Report/Appendix%207.pdf

(17) Barber, A. & Pellow, G. LCA: New Zealand Merino Wool Total Energy Use (AgriLINK NZ).

(18) Energy Technology System Analysis Programme. Hydrogen Production & Distribution. (IEAETSAP, **2014**).

(19) James, B. D., Houchins, C., Huya-Kouadio, J. M. & DeSantis, D. A. Final report: Hydrogen storage system cost analysis. (Strategic Analysis, Inc., 2016).

(20) Carbon Fiber and Global Environment | TORAYCA® | TORAY. Available at: https://www.torayca.com/en/aboutus/abo_003.html.

(21) Embodied Energy | Composites UK. Available at: https://compositesuk.co.uk/compositematerials/faqs/embodied-energy.

(22) Climate change - UKGBC - UK Green Building Council. Available at: https://www.ukgbc.org/climate-change/.

(23) Lauer, M. Methodology Guideline on Techno Economic Assessment (TEA) - Generated in the Framework of ThermalNet WP3B Economics (Joanneum Research, **2008**).

(24) Sara, H. R.; Enrico, B.; Mauro, V.; Andrea, D. C.; Vincenzo, N. Techno-economic Analysis of Hydrogen Production Using Biomass Gasification - A Small Scale Power Plant Study. *Energy Procedia* **2016**, *101*, 806–813.

(25) Vlysidis, A.; Binns, M.; Webb, C.; Theodoropoulos, C. A Techno-economic Analysis of Biodiesel Biorefineries: Assessment of Integrated Designs for the Co-production of Fuels and Chemicals. *Energy* **2011**, *36*, 4671–4683.

(26) Fisher, K.; Collins, M.; Aumônier, S.; Gregory, B. Carbon Balances and Energy Impacts of the Management of UK Wastes. Defra R&D Project WRT237. (ERM, **2006**).

(27) Standard rates | Anglian Water Services. Available at: https://www.anglianwater.co.uk/accountand-bill/tariffs-and-charges/standard-rates/.

(28) Mo, W.; Zhang, Q.; Mihelcic, J. R.; Hokanson, D. R. Embodied Energy Comparison of Surface Water and Groundwater Supply Options. *Water Res.* **2011**, *45*, 5577–5586.

(29) UK BOC Online Shop: Nitrogen (Oxygen Free) Cylinder. Available at: https://www.boconline.co.uk/shop/en/uk/nitrogen-oxygen-free-230-bar-cylinder#product1.

(30) Kaiser, J. A Comparison of Energy Use and Carbon Generated from the Operation and Maintenance of Passive Onsite and Centralized Wastewater Treatment Systems. Available at: https://www.infiltratorwater.com/Customer-Content/www/white-papers/PDFs/KAISER.pdf

(31) Liu, G.; Zhang, J.; Bao, J. Cost Evaluation of Cellulase Enzyme for Industrial-scale Cellulosic Ethanol Production Based on Rigorous Aspen Plus Modeling. *Bioprocess Biosyst. Eng.* **2016**, *39*, 133–140.

(32) Dunn, J. B.; Mueller, S.; Wang, M.; Han, J. Energy Consumption and Greenhouse Gas Emissions from Enzyme and Yeast Manufacture for Corn and Cellulosic Ethanol Production. *Biotechnol. Lett.* **2012**, *34*, 2259–2263.

(33) Dick, H.; Scholes, P. Gate Fees 2017/18 Final Report: Comparing the Costs of Alternative Waste Treatment Options. (WRAP, **2018**).

(34) ARUP & De Montfort University. Measuring Scope 3 Carbon Emissions - Water and Waste, **2012**.

(35) Hazardous Waste Disposal Costs for Businesses - Boulder County. Available at: https://www.bouldercounty.org/environment/hazardous-waste/disposal-costs-for-businesses/.

(36) Kaiser, J. A Comparison of Energy Use and Carbon Generated from the Operation and Maintenance of Passive Onsite and Centralized Wastewater Treatment Systems. (Infiltrator Water Technologies, **2017**).

(37) Caustic Soda Prices in USA, Europe, Asia. 200071 (2018). Available at: https://www.intratec.us/chemical-markets/caustic-soda-prices.

End of Supporting Information
